# Improving the scientific rigour of nutritional recommendations for adults with type 2 diabetes: A comprehensive review of the American Diabetes Association guideline‐recommended eating patterns

**DOI:** 10.1111/dom.13736

**Published:** 2019-05-09

**Authors:** Sarah J. Hallberg, Nancy E. Dockter, Jake A. Kushner, Shaminie J. Athinarayanan

**Affiliations:** ^1^ Medically Supervised Weight Loss Indiana University Health Arnett Lafayette Indiana; ^2^ Research Virta Health San Francisco California; ^3^ Department of Medicine Indiana University School of Medicine Indianapolis Indiana; ^4^ Independent Consultant Mabelvale Arkansas; ^5^ McNair Medical Institute and McNair Interests Houston Texas

**Keywords:** DASH, eating patterns, low‐carbohydrate, Mediterranean, plant‐based, type 2 diabetes

## Abstract

**Aims:**

The global rate of type 2 diabetes (T2D) continues to rise. Guidelines that influence the worldwide treatment of this disease are central to changing this trajectory. We sought in this review to evaluate the appropriateness of sources cited in the American Diabetes Association's (ADA) guidelines on eating patterns for T2D management, identify additional relevant sources, and evaluate the evidence.

**Materials and Methods:**

We reviewed the evidence behind the ADA's recommendations on eating patterns in the 2018 and 2019 ADA Standards of Care and the 2014 ADA Nutrition Therapy Recommendations for Adults with Diabetes. Additionally, we conducted a comprehensive search to identify any additional studies not included in the cited evidence. To determine appropriateness of inclusion in the guidelines, the following criteria were applied: 1) it was a clinical trial or systematic review/meta‐analysis of clinical trials; 2) it involved persons with T2D; 3) one of the study arms followed one of the eating patterns currently recommended; 4) its reported outcomes included glycaemic control; 5) outcomes were reported separately for persons with T2D.

**Results:**

We found a wide variation in the evidence for each eating pattern. Issues that have hampered the guideline process include: lack of a rigorous literature review, resulting in the omission of pertinent studies; an overreliance on prospective cohort studies; inconsistent standards for evidence; inclusion of studies not on persons with T2D; and bias.

**Conclusions:**

The ADA Guidelines recommended eating patterns fall short of rigorous standards of scientific review according to state‐of‐the‐art systematic review and guideline creation practices.

## 1 INTRODUCTION

Clinical practice guidelines are not new, but they are growing in number. A modern definition of clinical practice guidelines was set forth in 1992 by the Institute of Medicine and updated in 2011: “Clinical practice guidelines are statements that include recommendations intended to optimize patient care that are informed by a systematic review of evidence and an assessment of the benefits and harms of alternative care options.”^1^ Rising alongside the number of clinical practice guidelines are concerns about the process behind their creation. To ensure that guidelines affecting clinical care are created using the most rigorous and unbiased methods possible, multiple organizations have issued standards for evaluating scientific evidence when creating guidelines (Table S1). Despite the availability of standards to improve the development of clinical practice guidelines, there is still wide concern among the scientific community that even the most well‐respected guidelines lack sufficient rigour.^2^, ^3^, ^4^, ^5^, ^6^

Over half of adults in the United States now have type 2 diabetes (T2D) or prediabetes,^7^ and worse, this multifactorial epidemic is now worldwide and shows no signs of slowing, with rates of both T2D and T2D‐related health complications rising.^8^ When advising people with T2D on food choices, many healthcare providers rely on nutrition guidelines provided by the American Diabetes Association (ADA), and these guidelines influence standard recommendations made around the globe.^9^, ^10^, ^11^ Given these alarming trends, it is of paramount importance to review the treatment guidelines to ensure they are based on rigorous, accepted scientific methods.

The ADA's approach to the evidence in developing its guidelines has been to employ a grading system to rate the strength of evidence. An “A” rating is given to well‐conducted randomized controlled trials (RCTs) that are adequately powered, as well as to meta‐analyses that incorporate quality ratings. “B” ratings are given to well‐conducted cohort studies, “C” ratings are for poorly controlled trials or uncontrolled studies, and a score of “E” is for expert consensus or clinical experience. This approach does not follow any of the widely accepted standards or “guidelines for guidelines” such as Agree II, GRADE, or those from the National Academy of Sciences, Engineering and Medicine (Table S1).

Several concerns prompted our review of the evidence cited by the ADA in support of its recommendations for eating patterns in the management of T2D: (a) a strong reliance by the ADA on sources that they rate as B, C and E^12^, ^13^; (b) the failure to conduct a systematic review to inform source selection; (c) the exclusion of studies that could have been considered; (d) the lack of explanation of how the ADA selected and reviewed cited studies or how the experts weighed various endpoints in forming their opinion; and (e) the possibility of bias.

We conducted a review of the sources cited for currently recommended eating patterns in the ADA's Standards of Medical Care in Diabetes (Table 1) (2018 and 2019 standards),^12^, ^14^ and the ADA's Nutrition Therapy Recommendations for Adults with Diabetes (2014 recommendations),^13^ which helped inform the 2018 standards. In addition, a comprehensive search was conducted to identify any studies that would have been appropriate to include in a rigorous review. The review considers the strength of the evidence but does not assign a grade to each study.

**Table 1:** Description of eating patterns as described in the American Diabetes Association 2014 recommendations

Eating pattern	Description
**DASH diet**	Emphasizes fruits, vegetables, and low‐fat dairy products, including whole grains, poultry, fish and nuts, and is reduced in saturated fat, red meat, sweets and sugar‐containing beverages. The most effective DASH diet was also reduced in sodium.
**Mediterranean diet**	Includes abundant plant food (fruits, vegetables, breads, other forms of cereals, beans, nuts and seeds); minimally processed, seasonally fresh, and locally grown foods; fresh fruits as the typical daily dessert and concentrated sugars or honey consumed only for special occasions; olive oil as the principal source of dietary lipids; dairy products (mainly cheese and yogurt) consumed in low to moderate amounts; fewer than 4 eggs/wk; red meat consumed in low frequency and amounts; and wine consumption in low to moderate amounts generally with meals.
**Plant‐based diet** a	The two most common ways of defining vegetarian diets in the research are vegan diets (diets devoid of all flesh foods and animal‐derived products) and vegetarian diets (diets devoid of all flesh foods but including egg [ovo] and/or dairy [lacto] products). Features of a vegetarian‐eating pattern that may reduce risk of chronic disease include lower intakes of saturated fat and cholesterol and higher intakes of fruits, vegetables, whole grains, nuts, soy products, fibre and phytochemicals.
**Low‐carbohydrate** b	Focuses on eating foods higher in protein (meat, poultry, fish, shellfish, eggs, cheese, nuts and seeds), fats (oils, butter, olives, avocado), and vegetables low in carbohydrate (salad greens, cucumbers, broccoli, summer squash). The amount of carbohydrate allowed varies with most plans allowing fruit (eg, berries) and higher carbohydrate vegetables; however, sugar‐containing foods and grain products such as pasta, rice, and bread are generally avoided. There is no consistent definition of “low” carbohydrate. In research studies, definitions have ranged from very‐low‐carbohydrate diet (21‐70 g/d of carbohydrates) to moderately low‐carbohydrate diet (30%‐40% of calories from carbohydrates).

a More recently have been referred to as plant‐based diets but defined as vegetarian and vegan in the 2014 recommendations.

b More widely understood that this diet is not high in protein.

After this review was initially conducted, the ADA published their 2019 standards.^14^ In this new document, low‐carbohydrate diet was endorsed as a recommended eating pattern (new in 2019), with specific acknowledgment of the evidence for antiglycaemic medication reduction in people with T2D who adhere to a low‐carbohydrate diet. The present updated review includes all new citations from the 2019 ADA standards.

## 2 MATERIALS AND METHODS

The present review includes studies newly cited in the 2019 standards (Tables S2–S5), as well as sources cited in the 2014 recommendations and the 2018 standards (Table S7).

First, we sought to determine if each ADA‐cited study was appropriate for inclusion in the guidelines. Studies were deemed appropriate if: (a) they were a clinical trial, a systematic review, or a systematic review with meta‐analysis of clinical trials; (b) they involved people with T2D; (c) they had a study arm that followed one of the three eating patterns recommended by the 2018 standards or a low‐carbohydrate diet (low‐carbohydrate studies should specifically address quantity of carbohydrates); (d) their reported outcomes included glycaemic control; (e) their outcomes were reported separately for people with T2D if there was a T2D subgroup within a larger trial. Adherence to these criteria helps ensure that each included study belongs in the evidence base supporting the “cornerstone” of T2D management, which the ADA defines as metabolic control. The exclusion of prospective studies from our criteria was based on the judgement that such studies, while perhaps appropriate for T2D prevention guidelines, are not appropriate as a basis for treatment guidelines because they do not test a specific therapeutic intervention. In our review, we considered glycated haemoglobin (HbA1c) to be the primary biomarker for glycaemic control; fasting blood glucose (FBG) was considered if HbA1c data were not available. We also reported outcome data on lipids and lipoproteins, blood pressure and body weight, as these biomarkers are relevant for assessing overall cardiovascular disease (CVD) risk status, a critical component of T2D management.

Second, we searched the literature for other articles that might be appropriate for consideration in the development of dietary guidelines for T2D, following the same criteria by which we appraised studies cited by the ADA. The searches were performed in the following databases: PubMed and Medline Ovid. The searches were limited to human studies published in English between 1 January 2000 and 31 May 2018. We used the following search terms and/or combinations of these terms: diabetes; DASH; Dietary Approaches to Stop Hypertension; Mediterranean; vegetarian; vegan; plant‐based; low‐carbohydrate; carbohydrate restriction; carbohydrate‐restricted; and ketogenic. We also found other articles by reviewing references cited in relevant studies. A flow diagram of the search can be found in Figure S1.

Two co‐authors independently conducted the searches and evaluated all studies for appropriateness. In cases of disagreement, the two co‐authors and a third co‐author discussed the findings and reached agreement. All studies deemed appropriate for inclusion are presented in Tables S2–S5.

Third, we evaluated the evidence from all of the assembled studies, those cited by the ADA (Table S7) as well as those we had identified (Tables S2–S5). We did not assign a grade to each study but rather, on a prima facie basis, assessed whether or not the cited study provided evidence of benefit.

## 3 RESULTS

### 3.1 Dietary approaches to stop hypertension diet

#### 3.1.1 Cited evidence

The ADA 2014 recommendations and 2018 standards cite eight studies^15^, ^16^, ^17^, ^18^, ^19^, ^20^, ^21^, ^22^ (Table S7) to support claims that the Dietary Approaches to Stop Hypertension (DASH) diet is a healthy eating pattern for glycaemic control, blood pressure and other CVD risk factors in people with T2D. The cited studies include four RCTs: only one^15^ of the four RCTs^15^, ^16^, ^18^, ^19^ cited by the ADA was conducted in people with T2D. That study reported significant improvements in weight, FBG, blood pressure, HDL cholesterol, LDL cholesterol and HbA1c, but the trial was short (8 weeks), had a 30% dropout rate,^15^ and resulted in a 14.4% increase in triglyceride levels. The findings of two other ADA‐cited RCTs from the same study and published in two different journals showed significant reductions in systolic and diastolic blood pressure in the DASH study arm^16^, ^19^; however, neither study provided a sub‐analysis in people with T2D. The other four studies cited are an observational study,^20^ a commentary,^22^ a non‐systematic review,^21^ and the 2010 USDA Dietary Guidelines for Americans,^17^ which either reported a low incidence of T2D in those following the DASH diet or recommended the diet for blood pressure control.

#### 3.1.2 Additional evidence

We identified a post hoc analysis of the Exercise and Nutritional Interventions for Cardiovascular Health (ENCORE) study and an additional RCT,^23^, ^24^ both of which were published prior to the 2018 standards (Table S2). The post hoc analysis by Blumenthal et al^23^ compared a usual care diet, which allowed ad libitum energy intake, to the DASH diet alone and to a DASH diet with energy restriction and exercise. The DASH diet + exercise did result in significantly greater improvements in FBG, body fat, total cholesterol, LDL cholesterol and triglycerides compared to usual care, but the DASH diet alone did not have any of these significant outcomes compared to usual care. The study also reported a worsening in glycaemic control status (based on glucose tolerance test measures at baseline and end of the intervention) during the study period in participants without T2D or prediabetes in the DASH arm, more than with the control and DASH diet + exercise.^23^ The RCT by Paula et al^24^ compared the DASH diet + exercise to a diet based on ADA guidelines that did not include exercise. The significance of change from baseline and in a comparison of interventions was mixed; DASH + exercise resulted in a greater reduction in blood pressure but no difference in glycaemic control when compared to usual care; however, the effect of the DASH diet without exercise was unknown.^24^

#### 3.1.3 Summary of evidence

To our knowledge, clinical research on the DASH diet that provides outcomes for people with T2D consists of two RCTs, of 4 and 8 weeks' duration, and a post hoc analysis.^15^, ^23^, ^24^ Only one of the two trials showed glycaemic improvement that can be attributed to the DASH diet alone. According to our evaluation, the other cited sources provide limited to no support for the DASH diet for people with T2D in improving glycaemic control for the reasons already cited: these studies were not clinical trials or systematic reviews, or did not provide outcomes data for people with T2D. While evidence shows that the DASH diet reduces blood pressure, primarily in people without diabetes, the lack of evidence for glycaemic control does not support a recommendation for DASH as a healthy eating pattern for the management of T2D. Additionally, as can be seen in the other eating pattern sections, a decrease in blood pressure (critical for CVD risk management) can be achieved with other eating patterns with more robust glycaemic control data. To corroborate the current ADA recommendation for the DASH diet in management of T2D, more research is needed to closely evaluate the diet in those with T2D; particularly needed is research on glycaemic control and CVD risk factors as study endpoints.

### 3.2 Mediterranean diet

#### 3.2.1 Cited evidence

The ADA documents cite six studies,^25^, ^26^, ^27^, ^28^, ^29^, ^30^, ^31^ including three RCTs of longer duration,^25^, ^26^, ^27^, ^28^ to support claims that a Mediterranean diet can improve glycaemic control and CVD risk factors and is therefore a healthy eating pattern for people with T2D (Table S7). Two RCTs found that the Mediterranean diet was superior to comparison diets^25^, ^28^: one found that a low‐carbohydrate Mediterranean diet resulted in a significantly greater HbA1c reduction compared to the control diet,^28^ and the other found at 4‐year follow‐up that the Mediterranean diet resulted in significant HbA1c reduction, sustained improvements in triglyceride and HDL cholesterol levels, and less medication initiation in people with newly diagnosed T2D.^25^ A third RCT,^26^ for which data were reanalysed with essentially the same results in 2018,^27^ reported a significant reduction of major cardiovascular events in both versions of the Mediterranean diet studied, compared with the control. Two systematic reviews^29^, ^30^ found limited evidence that the Mediterranean diet is effective for glycaemic control, but more robust support for CVD risk reduction. Also cited was a commentary favouring the Mediterranean diet that was based on a non‐systematic selection of articles.^31^

#### 3.2.2 Additional evidence

We identified 12 other studies on the Mediterranean diet worthy of consideration: four RCTs, two RCT follow‐up studies, and six systematic reviews with meta‐analysis (Table S3).^32^, ^33^, ^34^, ^35^, ^36^, ^37^, ^38^, ^39^, ^40^, ^41^, ^42^, ^43^ One RCT found that this diet significantly improved HbA1c and body mass index in postmenopausal women with T2D, but the diet was not superior to usual care for improving blood pressure and lipids.^32^ A 2‐year RCT^36^ comparing low‐fat, low‐carbohydrate and Mediterranean diets in obese people with T2D, with data available for 36 persons with T2D, found that the Mediterranean diet improved FBG, but not HbA1c levels, compared to a low‐fat and low‐carbohydrate diet. Two studies^33^, ^34^ followed up Esposito 2009,^25^ which was included in the ADA‐cited evidence (Table 1). Both studies found longer times to medication requirement in the Mediterranean diet arm versus the low‐fat diet arm, as well as increased partial remission and improved FBG and CVD risk markers. One of two smaller 12‐week RCTs found a statistically significant HbA1c reduction favouring a Mediterranean diet over a typical diet; the other did not find a difference between the Mediterranean diet and a low‐fat diet.^35^, ^37^ Neither of these trials resulted in between‐group statistical significance for CVD risk factor markers including body mass index, blood pressure and lipids, but one found improvement in inflammation markers and flow‐mediated dilation in the Mediterranean diet arm only.^35^ Four systematic reviews with meta‐analysis^38^, ^39^, ^40^, ^41^ and two with network meta‐analysis^42^, ^43^ concluded that the Mediterranean diet is superior to other eating patterns for glycaemic control, weight loss, lipid profile, and reduced need for diabetes medication.

#### 3.2.3 Summary of evidence

The ADA‐cited sources combined with additional ones identified through our search resulted in a total of seven RCTs, two follow‐up RCT studies, and seven systematic reviews (including five with meta‐analysis) that are appropriate for consideration in developing nutrition guidelines for T2D. Among the included trials are several large‐scale studies, one with 3614 participants^26^, ^27^ and one with more than 200 participants.^25^, ^33^, ^34^ Longer‐term studies include one lasting 12 months,^28^ one lasting 24 months,^36^ and two lasting longer than 4 years.^25^, ^26^, ^27^, ^33^, ^34^

As recommended by the ADA guidelines, we found that the Mediterranean eating pattern has demonstrated effectiveness in improving glycaemic control^25^, ^28^, ^32^, ^33^, ^34^, ^38^, ^39^, ^40^, ^41^, ^42^, ^43^ as well as CVD risk factors and even in reducing CVD events.^22^, ^23^, ^26^, ^27^, ^29^, ^30^, ^33^, ^34^, ^38^, ^39^, ^40^, ^41^, ^42^, ^43^ This diet appears to be appropriately considered helpful for T2D management; its inclusion in the recommended eating patterns is warranted. However, questions remain about which components of the Mediterranean diet contribute to its effectiveness on all of these outcomes. Some studies suggest that it is the diet's more moderate carbohydrate content (<50% total energy intake) that accounts for reductions in weight and CVD risk,^44^ while others suggest that the high monounsaturated fat content in the diet plays an important role in improving insulin sensitivity, glycaemic control, and inflammation.^45^, ^46^ Research in these areas will strengthen future nutritional recommendations and provide more in‐depth guidance on how the Mediterranean diet can be used for T2D management.

### 3.3 Plant‐based diet

#### 3.3.1 Cited evidence

The ADA documents cite eight studies in support of a plant‐based diet^47^, ^48^, ^49^, ^50^, ^51^, ^52^, ^53^, ^54^ (Table S7) for glycaemic control and CVD risk reduction. Of three RCTs,^49^, ^51^, ^53^ none found a significant improvement in HbA1c over the control diet, although, in all three, the test diet resulted in reductions from baseline for HbA1c as well as diabetes medication use, a significant factor in the diet's overall effectiveness. In one RCT,^51^ a low‐fat vegan diet resulted in significantly greater FBG reduction than the control diet. The small study sample (11 total and four in the control arm) should be noted, as well as the lower energy intake prescribed for the vegan diet. Additionally, the follow‐up^54^ to the 2006 RCT by Barnard et al,^49^ which tested an energy‐controlled diet compared to an ad libitum vegan diet and initially found within‐group but not between‐group advantages for both diets, found a substantial decline in benefits occurring between 22 and 74 weeks; however, when the data were analysed before medication changes, a significant between‐group reduction in HbA1c was observed in the vegan group.^54^ In a review by Rinaldi et al^47^ whose conclusions favoured plant‐based diets, six trials did not consistently show improvements in glycaemic control, weight loss or CVD risk factors.^51^, ^52^, ^53^, ^55^, ^56^, ^57^ The ADA also cited a commentary based on a non‐systematic review,^48^ a cross‐sectional study,^52^ and an assessment of diets in Barnard et al^49^ 2006. None of these studies was a controlled trial or systematic review.

#### 3.3.2 Additional evidence

We identified nine studies^40^, ^56^, ^57^, ^58^, ^59^, ^60^, ^61^, ^62^ not included in the ADA review, three of which were published after the 2018 standards (Table S4). Three RCTs found reductions in HbA1c from baseline,^56^, ^57^, ^58^ and two found the test diet superior compared to the control diet.^56^, ^57^ In these studies, the plant‐based diets were compared to an energy‐restricted diet, the recommended Korean Diabetes Association diet, and the participants' usual diet. However, in all three studies, a slight increase in triglycerides was observed in the intervention arms, with one study reporting a statistically significant change.^57^ This study also reported significant decreases in weight, as well as in total, LDL and HDL cholesterol levels in the intervention arm.^57^ A follow‐up study^59^ to the 2011 study by Kahleova et al^53^ found that the significant improvements (from baseline) in HbA1c had regressed over time, even though the intervention arm maintained a significant weight loss and higher level of antiglycaemic medication reduction at 24 months. A single‐arm demonstration study^61^ found a plant‐based diet, coupled with digital support, was effective for glycaemic control, according to patient‐reported HbA1c outcomes, while another non‐randomized study found no significant change in glycaemic control compared to both baseline and the control diet.^57^ In addition, we found three systematic reviews with meta‐analysis. Yokoyama et al^61^ found that the evidence supports plant‐based diets for glycaemic control, but had left out the follow‐up study by Kahleova et al, while Ajala et al^40^ concluded that the evidence is only suggestive of benefit. Lastly, a systematic review with network meta‐analysis^43^ did not find plant‐based diets to be superior to other eating patterns for T2D.

#### 3.3.3 Summary of evidence

In summary, all six known controlled trials^9^, ^51^, ^53^, ^56^, ^57^, ^58^ and two follow‐up studies^54^, ^59^ showed improvements from baseline in HbA1c and FBG with a plant‐based diet; however, only two showed significant improvement compared to a control diet.^56^, ^57^ Longer‐term data from two follow‐up studies at 1 year and 74 weeks found no lasting significant benefit.^54^, ^59^ All controlled studies except one had fewer than 100 participants. Overall, as recommended by ADA guidelines, a plant‐based diet may be effective in improving glycaemic control for some people with T2D, especially in those with a personal preference for such an eating pattern, at least in the short term; however, some of the studies that showed improvements in glycaemic endpoints were restricted in energy intake^51^, ^53^; therefore, it is not clear exactly what generated the beneficial outcomes—the composition of the diet or the weight loss resulting from energy restriction.^63^, ^64^, ^65^ Further, the decrease in HDL cholesterol^57^, ^58^, ^66^, ^67^ and higher triglyceride levels^66^, ^67^ seen in some studies need to be considered. Whether these changes in CVD risk markers are clinically meaningful or associated with poor CVD outcomes needs to be closely assessed; any worsening in atherogenic dyslipidaemia, which has been found to indicate worsening insulin resistance status,^68^ needs to be weighed against the improvements in other aspects of the lipid profile. This may allow individualized recommendations based on values prior to diet initiation and to any changes in the lipid panel in response to a plant‐based diet.

### 3.4 Low‐carbohydrate diet

#### 3.4.1 Cited evidence

The ADA documents cite 19 studies (Table S7) in their review of low‐carbohydrate diets.^28^, ^29^, ^36^, ^69^, ^70^, ^71^, ^72^, ^73^, ^74^, ^75^, ^76^, ^77^, ^78^, ^79^, ^80^, ^81^, ^82^, ^83^, ^84^ Of the 14 RCT trials cited, one^72^ was inappropriately included, as noted in Table 1. Of the remaining 13 RCTs, five found a significant between‐group advantage for the low‐carbohydrate arm for glycaemic control.^28^, ^69^, ^71^, ^83^, ^84^ Of the eight that did not show a between‐group glycaemic advantage, all but one found a reduction from baseline, and three had greater reductions in medication use.^73^, ^74^, ^82^ Of the seven trials with a duration of ≥1 year, three showed sustained clinically significant improvements in HbA1c at 1 year,^28^, ^69^, ^82^ and two showed sustained meaningful benefit at 2 years.^36^, ^78^ Another 1‐year study found the low‐carbohydrate diet resulted in decreased glucose variability, which has been found to be an independent CVD risk factor, making it an important overall consideration.^85^ An isocaloric trial found the low‐carbohydrate arm had a significant decrease in insulin and visceral fat accumulation compared to a high‐carbohydrate arm.^70^

Of the 10 studies that reported on lipids, five found significant improvements in triglycerides with a low‐carbohydrate diet^74^, ^78^, ^82^, ^83^, ^84^; none resulted in a worsening. Six^28^, ^70^, ^71^, ^73^, ^74^, ^82^ of 10 studies reporting HDL cholesterol or total cholesterol:HDL cholesterol ratio found that the low‐carbohydrate diet resulted in significantly better outcomes than comparison diets; the others found nonsignificant differences between diets.^75^, ^78^, ^83^, ^84^ Seven of eight studies reporting LDL cholesterol found nonsignificant differences between diets,^71^, ^73^, ^75^, ^78^, ^82^, ^83^, ^84^ with four of them reporting a nonsignificant decrease in LDL cholesterol^78^, ^82^, ^83^, ^84^ in the low‐carbohydrate arm, while the other three studies had a nonsignificant LDL cholesterol increase in the low‐carbohydrate arm.^71^, ^73^, ^75^ One study found superior improvement with a low‐carbohydrate diet.^28^ Four systematic reviews with meta‐analysis cited by the ADA concluded that there is evidence supporting the use of low‐carbohydrate diets in patients with T2D,^29^, ^77^, ^79^, ^80^ although benefits were found in some cases to decline over time or with higher carbohydrate intake. A fifth non‐systematic review of meta‐analyses by van Wyk et al^80^ concluded that adherence may be the most significant barrier to efficacy with a low‐carbohydrate approach to glycaemic control.

#### 3.4.2 Additional evidence

We identified 27 additional studies: 10 RCTs (nine new, one follow‐up), 12 non‐randomized trials (11 new, 1 follow‐up), and five systematic reviews with meta‐analysis. Of these 27 additional evidence sources, 20 were published in time for inclusion in the 2014 recommendations, and 21 were published prior to the 2018 standards (Table S5).^86^, ^87^, ^88^, ^89^, ^90^, ^91^, ^92^, ^93^, ^94^, ^95^, ^96^, ^97^, ^98^, ^99^, ^100^, ^101^, ^102^, ^103^, ^104^, ^105^, ^106^, ^107^, ^108^, ^109^, ^110^ All 27 studies reported outcomes data for people with T2D and thus were appropriate for consideration in the development of nutritional recommendations for T2D management. Of the 10 RCTs, all of which reported on glycaemic control, nine found that a low‐carbohydrate diet resulted in a significant change from baseline to end of study^86^, ^87^, ^89^, ^90^, ^92^, ^93^, ^94^, ^96^; six also found a superior between‐group reduction favouring the low‐carbohydrate diet.^86^, ^87^, ^90^, ^92^, ^94^, ^96^ While some studies found that the control diet also improved glycaemic control significantly from baseline, none found the control diet superior to the low‐carbohydrate diet. All 12 single‐arm and non‐randomized trials found that a low‐carbohydrate diet significantly improved glycaemic control from baseline to end of study; the two studies that made between‐group comparisons found the low‐carbohydrate diet superior to the control diet.^99^, ^101^ We identified eight longer‐term studies (1‐3 years' duration),^86^, ^88^, ^91^, ^93^, ^97^, ^98^, ^99^, ^105^ of which five^86^, ^97^, ^98^, ^99^, ^105^ found significant glycaemic benefit sustained with a low‐carbohydrate diet; these include two 2‐year trials^97^, ^105^ and a 3‐year trial.^98^ Another longer trial also found sustained improvement in glycaemic control at 44 weeks.^103^ All of these studies assessed HbA1c as the primary glycaemic marker, 86, 88, 91, 93, 97, 98, 99, 103 except the study by Dashti et al,^105^ which only reported FBG.

Of 11 studies that reported on diabetes medication use,^84^, ^88^, ^89^, ^90^, ^91^, ^92^, ^97^, ^99^, ^103^, ^104^, ^106^ eight reported more medication reductions and/or elimination of glycaemic control medications in the low‐carbohydrate arm. Five of six studies that conducted between‐group comparisons of medication use found the low‐carbohydrate diet to be superior,^86^, ^88^, ^89^, ^91^, ^99^ and one study^92^ found that both diets reduced usage significantly from baseline with no between‐group difference. No study found the control diet to be superior although there was some reduction in medication use from baseline in two of the studies in the control group.^88^, ^90^

Overall a favourable result was seen with regard to triglycerides and HDL cholesterol. No study found the control diet to be superior or that a low‐carbohydrate diet significantly worsened triglycerides or HDL cholesterol. The additional evidence is mixed regarding the low‐carbohydrate diet's effects on LDL cholesterol. Eight studies found no significant change within group from baseline,^87^, ^88^, ^89^, ^91^, ^93^, ^95^, ^96^, ^106^ whereas five other studies found that the diet resulted in significant improvement^101^, ^105^, ^107^ or showed superiority to a control diet.^97^, ^98^ In another study, the diet improved LDL cholesterol significantly in women but not in men.^102^ Two studies found that the diet resulted in significant worsening from baseline.^99^, ^100^ However, the study by Hallberg et al^99^ reported no change between the test and control diets for measured apolipoprotein B, probably more pertinent to CVD risk than the calculated LDL cholesterol value, which is impacted proportionately by the significant rise in HDL cholesterol and decrease in triglycerides in the Friedewald equation used to calculate LDL cholesterol.^99^

Three of four additional systematic reviews,^40^, ^108^, ^110^ including two published since 2017,^40^, ^109^ recommended a low‐carbohydrate diet for T2D management, while one found no advantage with a low‐carbohydrate diet.^109^ A fifth systematic review, with network meta‐analysis, concluded that a low‐carbohydrate diet was superior for HbA1c reduction compared to other eating patterns, but that a Mediterranean diet was superior for reduction of FBG.^43^

#### 3.4.3 Summary of evidence

The studies that we deemed appropriate for consideration in the development of nutritional guidelines in T2D treatment consisted of 18 from the ADA review (one was a follow‐up study) and 27 from our search (two were follow‐up studies). These 42 separate studies included 22 randomized trials, 10 non‐randomized trials and 10 systematic reviews, eight of which included a meta‐analysis. Ten of the trials had >100 participants,^28^, ^73^, ^74^, ^76^, ^78^, ^90^, ^91^, ^97^, ^99^, ^101^ and 16 provided longer‐term data: 10 studies lasting 1 to 2 years,^28^, ^69^, ^73^, ^76^, ^84^, ^92^, ^94^, ^99^, ^103^, ^105^ five studies lasting 2 years,^36^, ^78^, ^88^, ^91^, ^97^ and one study providing follow‐up data at 3 years. Of six studies lasting ≥2 years,^36^, ^78^, ^88^, ^91^, ^97^, ^98^ five sustained a clinically meaningful HbA1c reduction (of at least 0.7% from baseline). Three of the four 2‐year studies reporting on diabetes medication use found significant reductions with a low‐carbohydrate diet compared to a control diet^88^, ^91^, ^97^; this includes the one study that did not sustain HbA1c reduction at 2 years.^88^

Evidence from 30 trials and 10 follow‐up studies shows that a low‐carbohydrate diet is an effective dietary approach for addressing dyslipidaemia. More than half of the studies that reported triglyceride levels found a significant improvement from baseline with a low‐carbohydrate diet; eight also showed superiority over a control diet.^28^, ^71^, ^84^, ^91^, ^96^, ^99^, ^101^, ^103^ Similarly, the evidence consistently showed significant improvements in HDL cholesterol with a low‐carbohydrate diet, with 10 studies finding a significant increase over control diet.^28^, ^70^, ^71^, ^73^, ^88^, ^89^, ^91^, ^99^, ^101^, ^103^ It is also worth again noting that two^99^, ^100^ studies showed a significant increase in LDL cholesterol in the low‐carbohydrate arm; the rest of the studies found no change or a decrease of LDL cholesterol. Adding a clause in future guidelines on monitoring LDL cholesterol or apolipoprotein B would further guide physicians in recommending this diet for their patients to ensure no additional CVD risk factor worsening, as individual results may vary.

The authors of the ADA guideline documents, in their evaluation of a low‐carbohydrate eating pattern, raise concerns about the quality of evidence that they do not apply to other dietary patterns. For example, regarding low‐carbohydrate diets, the 2014 recommendations state, “many of the studies were small, were of short duration, and/or had low retention rates.” However, these issues could apply to plant‐based and DASH eating patterns as well. Another concern, raised in the 2018 standards, is that there is “not a standard definition” of low‐carbohydrate diets. While we agree that this is important, the issue—which essentially centres on the question of what an efficaciously low‐carbohydrate intake level is—can be evaluated within the currently available literature. This approach was used in the meta‐analysis by Snorgaard et al in 2017, which showed that the lower the actual percentage of daily calories consumed as carbohydrate (as reported by research participants), the greater the glycaemic control achieved.^79^ One of the key limitations observed in most studies on low‐carbohydrate diet is the discrepancy between the prescribed and actual carbohydrate intake. Most participants end up consuming more carbohydrate at the end of the intervention than was prescribed, probably affecting the outcome. This is a limitation that can be seen with any dietary intervention for which the prescribed diet and the diet actually consumed tend to be very different. Another limitation is how many of the glycaemic control improvements were attributable to the specific dietary intervention and how many were attributable to the weight loss alone. This is an issue with any of the dietary patterns that resulted in weight loss and is an important area of future research.

## 4 SUMMARY

Treatment guidelines must be based on rigorous scientific standards that are consistently applied in order to ensure that guidelines are both reliable and credible. In reviewing the evidence cited in support of the ADA recommendations on eating patterns for T2D management, we found multiple reasons for concern. Although the ADA does provide a rubric for grading studies to include in its evidence review, not apparent in the 2018 or 2019 standards or the 2014 recommendations is a description of the process used to guide final selection decisions. Perhaps that is the source of the issues we find concerning; for example, studies were cited as evidence that by the ADA's own rubric were not A‐rated sources or that were not conducted in people with T2D, were not clinical trials, or were not based on a systematic review of the evidence.

Our literature searches added considerably to the body of credible evidence worthy of consideration for a thorough review of the ADA recommendations on eating patterns. We found two additional studies to include on the DASH diet, 12 studies on the Mediterranean diet, nine on plant‐based diets, and 27 on low‐carbohydrate diets. Almost all of these additional studies were published prior to the documents reviewed in the present paper.

We would like to note several things in the ADA documents that could be interpreted as evidence of bias, one of which is the inclusion of opinion pieces or reviews favouring the DASH, plant‐based and Mediterranean eating patterns that were not based on a systematic approach to the literature.^22^, ^47^, ^48^ Further, there seemed to be inconsistency in the ADA's determination of what constitutes sufficiently ample and rigorous evidence for its recommendations. For example, regarding glycaemic control, the ADA recommends the DASH diet on the basis of a single trial in T2D. For plant‐based diets, the ADA recommends on the basis of three trials and one follow‐up study, none of which showed superiority of the test diet over the control diet in HbA1c reduction^49^, ^51^, ^53^, ^54^ and despite its conclusion that vegetarian and low‐fat vegan studies “did not consistently improve glycaemic control or CVD risk factors except when energy intake was restricted, and weight was lost.” In contrast, the 2014 recommendations and both the 2018 and 2019 standards raise concerns about lack of sustainability with a low‐carbohydrate diet over the long term. While adherence is a common behaviour change problem, it is not unique to low‐carbohydrate diets, and the long‐term data on this approach are supportive.

Our review is based only on studies in which glycaemic control in people with T2D is an endpoint, because of its central importance to T2D management. The aim has been to produce a review and presentation (Tables S1 and S2) of a more complete body of evidence that is objective, fair and easily accessible to most readers and may prove useful in the creation of future iterations of the ADA guidelines.

Another section of the ADA guidelines on HbA1c target guidance was recently reviewed and assessed by the American College of Physicians when they issued new HbA1c target guidance. Using the Agree II instrument for evaluation, the American College gave a score of 3.7 out of 7 for the ADA guidelines, the second‐lowest of six guidelines scored. Additionally, the ADA guidelines scored significantly lower than all others in “rigor of development.” Table S6 provides our assessment of the ADA guidelines using the National Academies of Sciences, Engineering, and Medicine's *Clinical Practice Guidelines We Can Trust* evaluation method*,* along with recommended steps for improving the overall process. Additionally, another review evaluated the evidence for CVD prevention in the 2016 edition of the Standards of Care.^4^ The prior two and current reviews of separate sections of the ADA guidelines all raise the same underlying concern regarding the rigour of the guideline development process. Given this, we believe our review is a critically important document that reinforces the need for a process change.

## 5 CONCLUSION

In order to change the current global trajectory of T2D, it is imperative that health organizations be willing to invest resources in creating objective guidelines based on rigorous and unbiased scientific review. Guidance from the ADA is valuable on many fronts; however, the present review of the current standards and recommendations, specifically on recommended eating patterns, finds significant shortcomings regarding scientific review methodologies, which are likely to translate to suboptimal clinical care decisions for people with T2D.

## CONFLICT OF INTEREST

S.J.H. is an employee and shareholder of Virta Health, a for‐profit company that provides remote diabetes care using a low‐carbohydrate nutrition intervention, and serves as an advisor for Atkins Corp. N.E.D. is a paid consultant for Virta Health. J.A.K. serves as medical director of McNair Interests, a private equity group with investments in type 1 diabetes and other chronic illnesses, and is also an advisor for Sanofi and Lexicon. S.J.A. is an employee and shareholder of Virta Health.

## Supporting information


**Figure S1.** Search flow diagram.
**Table S1.** Summary of additional studies on DASH diet worthy of consideration.
**Table S2.** Summary of additional studies on Mediterranean diet worthy of consideration.
**Table S3.** Summary of additional studies on plant‐based diets worthy of consideration.
**Table S4.** Summary of additional studies on the low‐carbohydrate diet worthy of consideration.
**Table S5** Standards for reviewing scientific evidence for clinical guidelines.
**Table S6.** Assessment and recommendations for improvement of the ADA guidelines using the National Academies of Sciences, Engineering and Medicine's *Clinical Practice We Can Trust* evaluation method.
**Table S6.** Assessment and recommendations for improvement of the ADA guidelines using the National Academies of Sciences, Engineering and Medicine's *Clinical Practice We Can Trust* evaluation method.
**Table S7** Summary of evaluation of studies on different eating patterns cited in the American Diabetes Association 2018 guidelines and 2014 nutrition recommendations
