# Supplementary material for: Improving the scientific rigour of nutritional recommendations for adults with type 2 diabetes: A comprehensive review of the American Diabetes Association guideline‐recommended eating patterns
Source: Diabetes Obes Metab. 2019 May 9;21(8):1769–79. doi: 10.1111/dom.13736 (PMC6767093; doi:10.1111/dom.13736)
Supplement: Supplementary file 1 — Figure S1. Search flow diagram. Table S1. Summary of additional studies on DASH diet worthy of consideration. Table S2. Summary of additional studies on Mediterranean diet worthy of consideration. Table S3. Summary of additional studies on plant‐based diets worthy of consideration. Table S4. Summary of additional studies on the low‐carbohydrate diet worthy of consideration. Table S5 Standards for reviewing scientific evidence for clinical guidelines. Table S6. Assessment and recommendations for improvement of the ADA guidelines using the National Academies of Sciences, Engineering and Medicine's Clinical Practice We Can Trust evaluation method. Table S6. Assessment and recommendations for improvement of the ADA guidelines using the National Academies of Sciences, Engineering and Medicine's Clinical Practice We Can Trust evaluation method. Table S7 Summary of evaluation of studies on different eating patterns cited in the American Diabetes Association 2018 guidelines and 2014 nutrition recommendations [file DOM-21-1769-s001.pdf]

Supplemental Figure 1

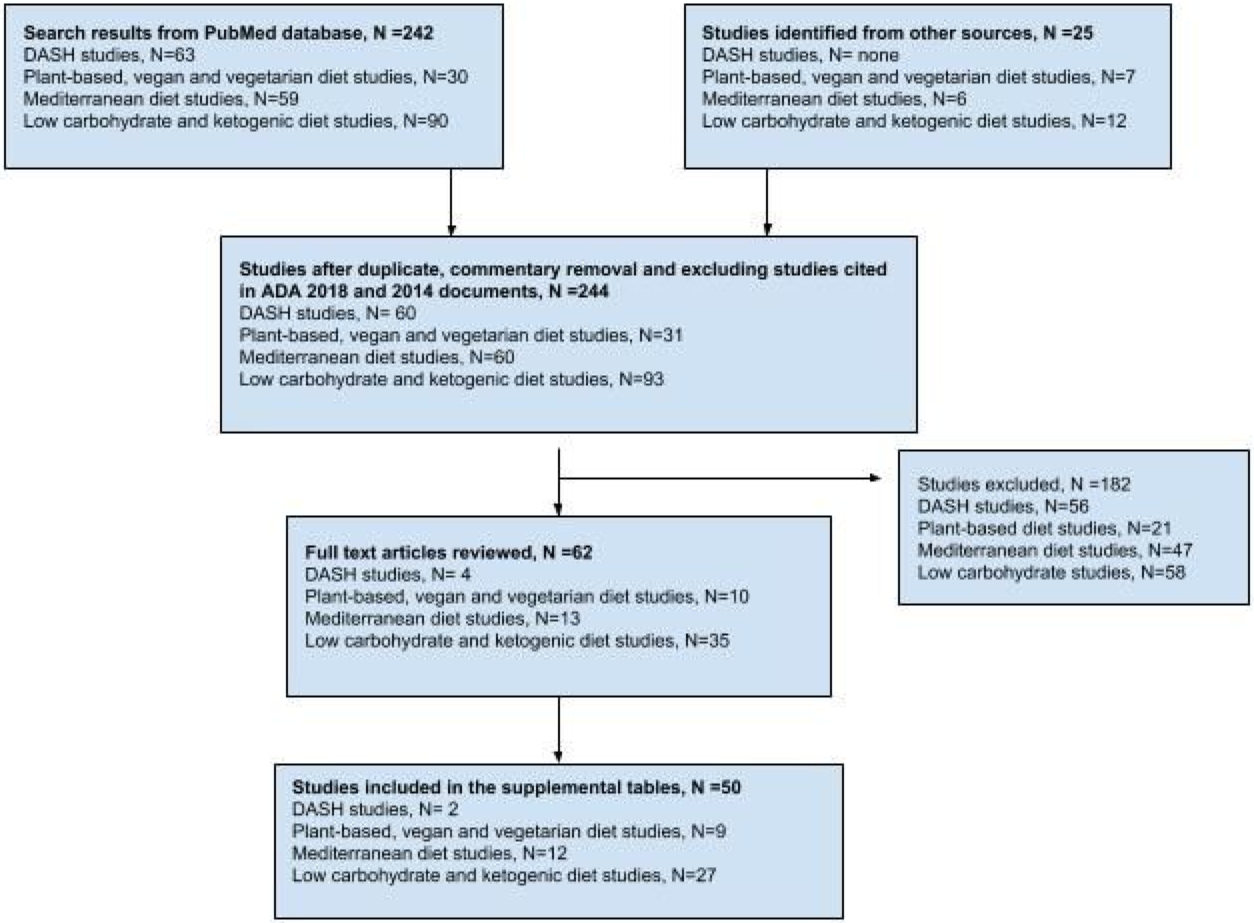

Supplemental Table S1. Summary of additional studies on DASH diet worthy of consideration

Study #	Citation	Study Type	Description	Subjects	Duration	Findings for DASH Diet	Comments (Does the study support claims of benefit from each eating pattern, overall?)
**1**	Blumenthal J, 2010[23]	RCT (ENCORE study)	Compared DASH; DASH + exercise + E- restriction; usual care	n=144 adults<br><br>140 (97%) completed study<br><br>n adults with prediabetes or T2D= 50 Completion n unknown	4 months	**In those with prediabetes and T2D:**<br>♦ % who improved in at least 1 category:<br>DASH + exercise/E-restriction – 72%<br>DASH alone – 54%<br>UC – 42%<br><br>♦ **In participants without diabetes or prediabetes at baseline - during the trial period diabetes classification worsened in:**<br>2% of DASH + exercise<br>16% in DASH alone<br>11% in UC<br>.	**Limited support for claims.**
**2**	Paula TP, 2015[24]	RCT	Compared DASH diet + exercise vs ADA-recommended diet + no exercise in adults with T2D and uncontrolled hypertension.	**n=**40 adults with T2D and high blood pressure<br><br>n that completed study is unknown	4 weeks	♦ **BP** DASH arm significantly improved.<br><br>♦ **HbA1c** both diets improved SS WG; BG nSS.<br><br>♦ **FBG** DASH only improved WG SS; BG nSS.<br><br>♦ **TG, LDL** DASH only improved WG SS; BG nSS.<br><br>♦ **Total C and HDL** nSS WG, nSS BG for both diets.<br><br>♦ **BMI** WG nSS, BG nSS	**Limited support for claims.**

Supplemental Table S2. Summary of additional studies on Mediterranean diet worthy of consideration

Study #	Citation	Study Type	Description	Subjects	Duration	Findings for Med Diet	Comments (Does the study support claims of benefit from each eating pattern, overall?)
1	Toobert D, 2003[32]	RCT	Med diet vs. control diet (usual care). No E-restriction. Included exercise and intensive behavioral support.<br><br>Main outcomes included HbA1c, lipids, BMI, blood pressure.	n=279 postmenopausal women with T2D<br><br>n that completed the study is unknown	6 months	**BG SS findings favored Med diet**<br><br>♦ **HbA1c** AR ↓0.36% (↓4.8%) BG SS<br><br>♦ **BMI** AR ↓0.37kg/m^2 (↓1.0%) BG SS<br><br>♦ **Lipids, blood pressure** BG nSS	**Supports claims.**
2	Maiorino, 2017[33]	RCT follow up	Follow up to Esposito 2009 and Esposito 2014. Assessed the long-term effects of a Mediterranean diet, compared to a low-fat diet, on circulating levels of endothelial progenitor cells (EPCs) and the carotid intima-media thickness (CIMT) in patients with T2D	n=215 adults with newly diagnosed T2D<br><br>101 (47.0%) completed study	End of trial varied with diet (based on mean years at which diabetes drugs needed)	**Results at EOT (mean follow-up 5.2 years Med diet; 3.5 years low-fat diet):**<br><br>♦ **CIMT** Med diet AR ↓0.026 mm (↓3.1%); low-fat diet AR ↓0.001 mm (↓.01%) BG SS<br><br>♦ **Circulating EPCs** On 7 measures, 5 were BG SS, favoring Med diet<br><br>♦ **HbA1c** Med diet AR ↓.7% (↓9.0%); low-fat AR .4% (5.0%) BG SS<br><br>♦ **HDL** Med diet AI 3mg/dL (↓7.0%); low-fat 0mg/dL (↓0%); BG SS<br><br>♦ **HOMA** Med diet AR ↓1.5 (↓28.8%); low-fat AR ↓.8 (↓15.1%); BG SS<br><br>♦ **Weight** Med diet AR ↓3.7kg (↓4.3%); low-fat diet AR ↓2.9kg (↓3.4%) BG SS<br><br>♦ **Total C, FBG** BG nSS	**Supports claims.**

						♦ **Mean years at which diabetes drugs needed** Med diet, 5.2 years; low-fat diet, 3.5 years.	
3	Esposito, 2014[34]	RCT follow up	Follow up to Esposito 2009, which tested Med diet for efficacy in delay of medication initiation in patients newly diagnosed with T2D.	Original n **=** 215 overweight persons newly diagnosed with T2D	End of trial varied with diet (based on mean years at which diabetes drugs needed)	♦ **Mean years at which diabetes drugs needed Med diet,** 4.8 years; low-fat diet, 2.8 years.<br><br>♦ **% experiencing T2D remission (partial or complete) at yr 6:** Med diet, 5.0%; low-fat diet, 0%.<br><br>♦ **% experiencing complete T2D remission at yr 4:** Med diet ,1.9%; low-fat diet 0%<br><br>♦ all Med diet participants required diabetes medications at 8.1 years. all low fat participants required medications at 6.1 years	**Partially supports claims.**
4	Ceriello, 2014[35]	RCT	Tested effects of a Med diet supplemented with olive oil vs a control low-fat diet	n=24 adults with T2D<br><br>n completed unknown	3 months	♦ **Flow-mediated dilatation (FMD)** Med diet AR ↓2.3% (↓29.1%) WG SS; low-fat, WG nSS.<br><br>♦ **In addition to FMD Med diet** SS improved 4 other markers for inflammation and reduced negative effects of acute hyperglycemia induced by hyperglycemic clamp. WG SS; low-fat diet WG nSS on all.<br><br>♦ **HbA1c, BMI, SBP, DBP, total C, TRG, HDL, LDL** WG nSS, BG nSS for both diets.	**Partially supports claims.**
5	Shai I, 2008[36]	RCT	Compared Med, low-carb, and low-fat diets.	n=322 moderately obese adults with T2D; included 46 adults with T2D<br><br>36 (78%) with T2D completed study	2 years	**In those with T2D:**<br><br>♦ **FBG** better with Med diet; BG SS (vs low-fat diet)<br><br>♦ **HOMA-IR** better with Med diet; BG SS (vs low-fat diet)<br><br>♦ **HbA1c**<br>Low-fat AR ↓0.4% WG nSS	**Supports claims.**

						Med AR ↓0.5% WG nSS<br>Low-carb AR ↓0.9% WG SS<br>BG nSS	
6	Itispoulos, 2010[37]	RCT	Investigated the impact on metabolic control of a diet modeled on the traditional Cretan Med diet in T2D	n=31<br><br>27 (87%) completed study	12 weeks	**For Med diet:**<br><br>♦ **HbA1c AR** ↓.3% (4.2%) BG SS for Med diet<br><br>♦ **BMI, FBG, fasting insulin, total C, HDL, LDL, TRG, SBP, DBP, CRP, HOMA** BG nSS	**Partially supports claims.**
7	Esposito, 2010[38]	Systematic review and meta-analysis	assessed the effect of the Med diet management and prevention of T2D.	Included 5 RCTs on persons with T2D	varied	♦ **FBG (4 RCTs)** AR range 7-40mg/dL. BG SS,favoring Med diet.<br><br>♦ **HbA1c** (4 RCTs) AR range 0.1-0.6%. BG SS favoring Med diet.<br><br>♦ **HOMA** (3 RCTs) favored Med diet.	**Supports claim**
8	Esposito, 2015 [39]	Systematic review and meta-analysis	Summarized the evidence about the efficacy of the Med diet on the management of T2D and prediabetic states.	8 meta-analyses and 5 RCTs included.		♦ **HbA1c from meta-analysis of 3 trials on T2D (≥ 6 mos, pooled n=673)** favored Med diet, WMD= -.47% BG SS<br><br>♦ **HbA1c from meta-analysis of 4 meta-analyses on T2D.** Favored Med diet, AR range ↓0.30-.47%.	**Supports claims.**
**9**	Ajala O, 2013[40]	Systematic review and meta-analysis	Assessed the effect of diet types on glycemic control, lipids, and weight loss. Based on search to Aug 2011 for RCTs of ≥ 6 mo. Includes 4 studies on Med diet.	Adults with T2D		Found the Med diet superior to other diets for glycemic control (HbA1c) and weight loss; improved TRG, HDL, need for diabetes medication.	**Supports claims.**
**10**	Huo R, 2015[41]	Systematic review and meta-analysis	Meta-analysis of RCTs to explore the effects of the Med diet, compared to control diets, on glycemic control, weight loss and CVD risk factors in persons with T2D. Included 9 studies. Based on search of literature to Feb 2014.	Adults with T2D		Med diet resulted in greater improvement in HbA1c, FBG, fasting insulin, blood pressure, weight loss, HDL and TRG than control diets.	**Supports claims.**

**11**	Pan B, 2018[42]	Systematic review/ network meta-analysis	Compared the differences between major dietary patterns (Mediterranean, low-carb, low-fat) in improving glycemic control,cardiovascular risk, and weight loss for patients with T2D	10 RCTs (2006 to 2016)		♦ Included studies were on Mediterranean, low-fat, low-carb, high-carb, and regular diets.<br><br>♦ The only BG SS mean differences: 1) high-carb superior to regular diet for HDL (1.04mmol/L MD); 2) Med diet superior to low-fat for HbA1c (-.45% MD); FBG (-1.24mmol/L MD); weight (-1.18kg MD); waist size (-0.73 cm MD); HDL (0.07mmol/L MD); total C (-0.17mmol/L MD); and TRG (-0.21mmol/L MD).<br><br>♦ No other between-diet comparisons found SS differences between diet types.<br><br>♦ The Med diet was ranked as having the highest probability of being the best diet for improving glycemic control (HbA1c), weight, waist circumference, CVD risk (LDL, total C, TRG**).**	**Supports claims.**
**12**	Schwingshackl L, 2018[43]	Systemic review and network meta-analysis	Compared the efficacy of different dietary approaches on glycemic control in T2D. Nine dietary approaches were included low-fat, vegetarian, Mediterranean, high-protein, moderate-carbohydrate, low-carbohydrate, control, low GI/GL, Paleothilic). Lit search through July 2017. 56 trials were included, published between 1978 and 2016.	Adults with T2D	$\geq$ 12 weeks	**For Med diet:**<br><br>♦ **HbA1c** AR↓0.79% vs control<br><br>♦ **Fasting glucose** AR↓1.61mmol/L vs control	**Supports claim**

Supplemental Table S3. Summary of additional studies on plant-based diets worthy of consideration

Study #	Citation	Study Type	Intervention	Subjects	Duration	Findings	Comments (Does the study support claims of benefit from each eating pattern, overall?)
**1**	Barnard N, 2018[55]	RCT	Tested efficacy of low-fat vegan diet vs. control diet in clinical setting. E-restricted only in control diet.	**n=**45 obese adults with T2D<br><br>40 (89%) completed study	20 weeks	**For plant based:**<br><br>♦ **HbA1c** AR ↓0.4% (↓6.0%) WG SS, BG nSS<br><br>♦ **Weight** AR ↓6.3kg (↓6.4%) WG SS, BG nSS<br><br>♦ **SBP, DBP** nSS<br><br>♦ **LDL** AR ↓11.9mg/dL (↓15.7%) WG SS, BG nSS<br><br>♦ **HDL** AR ↓3.4mg/dL (↓6.2%) WG SS, BG nSS<br><br>♦ **TRG** AR ↑20.8mg/dL (13.3%) WG nSS, BG nSS.	**Limited support for claims.**
**2**	Kahleova H, 2014[56]	RCT follow up	Follow up from Barnard 2006. Compared E-restricted vegetarian and conventional diabetic diets on body fat, IS, oxidative stress.	n=62 obese adults with T2D in original study<br><br>n at FU=44 (71%)	1 year after end of 6-month RCT	**For plant based:**<br><br>♦ **HbA1c** AI ↑0.49% WG SS, BG nSS<br><br>♦ **Diabetes medication use hypoglycemics AI** ↑14%; insulin introduced AI ↑5%	**Limited support for claims.**

						♦ **Weight** AR nSS at 1 yr (at 6 mo, AI ↑1.7kg, WG SS)<br><br>♦ **Lipids** nSS (WG SS improvement at 6 mo)<br><br>♦ Most of the positive results at end of original study and at 6-mo FU were not sustained at 1-yr FU.	
**3**	Lee Y, 2016[57]	RCT	Compared vegan diet to Korean Diabetes Assn. diet.	n=106 adults with T2D<br><br>93 (88%) completed study	12 weeks	**For vegan diet:**<br><br>♦ **HbA1c AR** ↓0.5% (↓6.5%) WG SS, BG SS favored vegan diet<br><br>♦ **Weight** AR BMI ↓0.5kg/m^2 (↓2.1%) WG SS, BG nSS<br><br>♦ **Lipids, blood pressure** nSS	**Supports claims.**
**4**	Mishra S, 2013[58]	Multi-site trial with sites randomized for test and control arms	Worksite program comparing no diet change to low-fat vegan diet for weight, lipids, blood pressure and HbA1c.	**n=**291 subanalysis of T2D n=43<br><br>35 (81%) with T2D completed study	18 weeks	**For plant-based with T2D:**<br><br>♦ **HbA1c** AR ↓0.6% (↓9.8%) WG SS, BG SS<br><br>♦ **Lipids not reported separately for T2D but for overall completers in plant based arm:**<br>♦ **TRG: AR** ↑13.9 (10%) WG SS<br><br>♦ **HDL: AR** ↓3.3 (9%) WG SS<br><br>♦ **LDL: AR** ↓13 (12%) WG SS<br><br>♦**Weight: AR** ↓4.3 (6%)	**Partially supports claims**
**5**	Ferdowsian H, 2010[59]	Non-randomized, two-site trial	To determine whether a multicomponent intervention program at a corporate site in is effective in reducing body weight and improving cardiovascular risk factors in overweight individuals.	n=113 adults with a BMI of kg/m^2 and/or previous diagnosis **of** T2D.<br><br>n with T2**D=**19; 100% completed	22 weeks	**For plant-based with T2D:**<br><br>♦ **HbA1c** AR ↓0.3% (↓4.1%) WG nSS, BG nSS<br><br>♦ Among those without medication changes (data not reported), **HbA1c** AR ↓0.9% (n=5) in the intervention vs AR ↓0.2% (n=6) in the control. SS not reported.	**Limited support for claims.**

**6**	Berman MA, 2018[60]	Single arm demonstration	Tested PB diet + exercise intervention that used digital therapeutics for glycemic control and medication use. HbA1c results were patient-reported.	n=118 adults with T2D<br><br>96 (81%) completed	12 weeks	♦ **HbA1c** AR↓ 0.8% (↓9.9%) SS	**Supports claims.**
**7**	Schwingshackl L, 2018[43]	Systemic review and network meta-analysis	Compared the efficacy of different dietary approaches on glycemic control in T2D. Nine dietary approaches were included low-fat, vegetarian, Mediterranean, high-protein, moderate-carbohydrate, low-carbohydrate, control, low GI/GL, Paleothilic). Lit search through July 2017. 56 trials were included, published between 1978 and 2016.	Adults with T2D	$\geq$ 12 weeks	♦ **HbA1c** AR↓0.67% vs control<br><br>♦ **Fasting glucose** AR↓1.29mmol/L vs control	**Supports claims.**
**8**	Yokoyama Y, 2014[61]	Systematic review and meta-analysis	Lit search 1900 through Dec 9 2013 for trials on PB diet in adults with T2D ≥ 4 weeks and reporting HbA1c and FBG. Six studies included: 4 RCTs and two clinical trials. A follow-up study was excluded which may have impacted findings	Adults with T2D	4-22 weeks	**HbA1c** Mean absolute difference between test and control diets was 0.39%. favored vegan diet	**Supports claims.**
**9**	Ajala O, 2013[40]	Systematic review and meta-analysis	Lit search to August 2011 of RCTs ≥ 6 mo comparing various diet types. Included 2 RCTs on PB diets (Barnard, 2009 and Kahleova, 2011); these two studies were not included in the meta-analysis.			"...there is a suggestion that vegan and vegetarian diets might be beneficial in improving glycemic control and inducing weight loss. However, there is a need for more studies to support the wider use of these diets in people with diabetes."	**Limited support for claims.**

Supplemental Table S4. Summary of additional studies on the low-carbohydrate diet worthy of consideration

Study #	Citation	Study Type	Description	Subject s	Duration	Findings for the Low-Carb Diet	Comments
**Randomized Controlled Trials**							
**1**	Saslow L, 2017[86]	RCT	Compared ad libitum low-carb, ketogenic diet vs. moderate-carb, lower-fat, E-restricted diet	n=34 adults with T2D<br><br>29 (85%) completed study.	1 year	**The BG SS findings favored low-carb.**<br><br>♦ **HbA1c** AR ↓0.5% (↓7.6%) BG SS WG SS for both<br><br>♦ **Weight** AR ↓7.9kg (↓7.9%) BG SS<br><br>♦ **Fasting insulin** BG nSS<br><br>♦ **HOMA2-IR** BG nSS<br><br>♦ **HDL, TRG, LDL** BG nSS<br><br>♦ **SBP, DBP** BG nSS<br><br>♦ **TRG:HDL ratio** AR ↓0.5 (↓22.7%) BG SS<br><br>♦ **Diabetes medication use** Sulfonylureas or dipeptidyl peptidase-4 inhibitors: all 6 in low-carb diet group discontinued the meds by 12 months. BG SS. No medications were discontinued in control group.<br><br>♦ Metformin: BG nSS	**Supports claims.**
**2**	Yamada Y, 2014 [87]	RCT	Examined the effects of a non-E-restricted, low-carb diet vs. an E-restricted diet.	n=24 adults with T2D<br><br>24 (100%) completed study.	6 months	**The BG SS finding favored low-carb.**<br><br>♦ **HbA1c** AR ↓0.6% (↓8.6%) BG SS<br><br>♦ **FBG** AR BG nSS<br><br>♦ **Weight** WG nSS, BG nSS<br><br>♦ **LDL, HDL, SBP, DBP** WG nSS, BG nSS	**Partially supports claims.**

						♦ **TRG** AR ↓58.2mg/dL (↓41.1%) WG SS BG nSS<br><br>**Safety and Adverse Effects**<br>"...no changes in the markers of the renal function (i.e., urinary nitrogen, Cr, eGFR and albumin-to-creatinine ratio) … in either group. A marker of the liver function, the alanine aminotransferase level, tended to improve in the low-carbohydrate group ..."	
**3**	Guldbrand H, 2012 [88]	RCT	Compared the effects on HbA1c and weight loss of a low-fat diet vs. a low-carb diet.	n=61 adults with T2D.<br><br>7 did not take part, but their outcomes data were included; 100% included in analysis.	2 years	♦ **HbA1c**<br>6 mo AR ↓0.4% (↓5.3%)<br>24 mo ↓0%<br>Low carb diet WG SS at 6 mos.<br>Low-fat diet WG nSS at all points.<br>BG nSS at 24 mos.<br><br>♦ **Weight** low carb WG SS AR at all points; ↓2.0kg (↓2.2%) at 24 mo. Low-fat diet WG SS AR at all points. BG nSS at 24 mo.<br><br>♦ **SBP and DBP** Both diet groups WG SS AR at all points., BG nSS at 24 mo.<br><br>♦ **HDL** WG SS AI at all points; ↑0.23mmol/L (↑20.4%) at 24 mo. BG SS. Low-fat diet WG SS worse at 12, 24 mo.<br><br>♦ **LDL AR** ↓0.3mmol/l WG nSS<br>♦ **Diabetes medication use**<br>Insulin doses ↓ BG SS. No clinically significant reduction in medications in the control group.	**Limited support for claims.**<br><br>.
**4**	Westman E, 2008 [89]	RCT	Compared a very low-carb, ketogenic diet vs. an E-restricted low-glycemic index diet.	n=84 obese adults with T2D<br><br>49 (58%) completed study.	24 weeks	**For low carbohydrate:**<br><br>♦ **HbA1c** AR ↓1.5% (↓17.0%) WG SS, BG nSS	**Supports claims.**

						♦ **FBG** AR ↓19.9mg/dl (↓11.2%) WG SS BG nSS<br><br>♦ **Weight** AR ↓11.1kg (↓10.2%) BG SS<br><br>♦ **Fasting insulin** AR ↓2.2uU/mL (↓29.4%) WG SS; BG nSS<br><br>♦ **HDL** AI↑ 5.6mg/dL (↑12.7%) WG SS, BG nSS<br><br>♦ **TRG** AR ↓67.5mg/dl (↓32.1%) WG SS, BG nSS<br><br>♦ **Total C**, **LDL** WG nSS, BG nSS<br><br>♦ **VLDL** AR ↓10.0mg/dl WG SS, BG nSS<br><br>♦ **TRG:HDL ratio** AR ↓1.8 (↓34.6%) WG SS, BG nSS<br><br>♦ **Diabetes medication use** 95.2% subjects reduced or eliminated meds. BG SS favoring low-carb. WG reduction in both arms.<br><br>♦ **Adverse events:** symptomatic; most common - headache, constipation, diarrhea, insomnia, and back pain BG nSS for all.	
**5**	Samaha F, 2003 [90]	RCT	To study the effects of a low-carb diet vs. a E-restricted, low-fat diet in severely obese individuals. inst to consume <30gr total of carbohydrates per day in low carbohydrate arm.	n=132 severely obese subjects most having dT2D or MetSyn; 79 (59.9%) completed the study. T2D sub-analysis conducted for FBG only.	6 months	**Results low carbohydrate in participants with T2D:**<br><br>♦ **FBG** AR ↓26.0 mg/dL (↓15.5%) BG SS favoring low-carb.<br>♦ **insulin level** AR ↓8 (20%) BG nSS<br>♦ **HbA1c** AR ↓0.6 (8%) BG nSS	**Supports claims.**
**6**	Tay J 2018 [91]	RCT follow-up	Follow up to Tay 2014. Compared a very low-carb,	**n=**115 obese adults with T2D	2 years	**The BG SS findings favored low-carb.**	**Partially supports claims**

			high-unsaturated/low-saturated fat diet vs. a high-unrefined carb, low-fat diet on glycemic control and CVD risk in T2D.<br><br>Both groups were E-restricted.	61 (53%) completed study.		♦ **HbA1c** ↓0.7% in both groups BG nSS<br><br>♦ **Glucose variability** Improved more in LC group BG SS<br><br>♦ **Weight** maintained almost 7% weight loss in both groups for 2 years BG nSS<br><br>♦ **TRG** AR ↓.1mmol/L (↓6.3%) BG SS<br><br>♦ **HDL** AI ↑0.02mmol/L (↑1.7%) BG SS<br><br>♦ **LDL** WG nSS, BG SS favoring control diet.<br><br>♦ **SBP, DBP, FBG, fasting insulin, HOMA2-IR, CRP, eGFR** BG nSS<br><br>♦ **Diabetes medication use** Reduced more in low-carb arm BG SS. WG reduction in both arms.	
**7**	Wang L, 2018 [92]	RCT	Compared a low-fat diet (recommended in China for T2D) to a low-carb diet for efficacy in control of blood glucose levels in persons with T2D	n=56 adults with T2D<br><br>49 (88%) completed study.	3 months	♦ **HbA1c** AR ↓.63% (↓8.5%) WG SS. Low-fat diet also WG SS. BG SS favoring low-carb.<br><br>♦ **FBG** AR ↓1.41mmol/L (↓17.0%) WG SS. Low-fat diet also WG SS. BG nSS.<br><br>♦ **BMI** AR ↓.77kg/m^2 (↓3.2%) WG SS. Low-fat diet WG nSS. BG nSS.<br><br>♦ **Total C** AR ↓.36mmol/L (↓7.4%) WG SS. Low-fat diet WG nSS. BG nSS. Other lipids not reported.<br><br>♦ **Diabetes medication use** Both diets ↓~3 insulin units/day. WG SS for both. BG nSS.<br><br>♦ **Adherence** same degree of adherence in both groups	**Supports claims.**

**8**	Larsen RN, 2011[93]	RCT	To compare high protein, low carbohydrate (30% energy from protein and 40% energy from carbohydrate) with low protein, high carbohydrate diet (15% energy from protein and 55% energy from carbohydrate)	n= 99 obese or overweight adults with T2D<br><br>93 (94%) completed the study	12 months	**All results for 40% carbohydrate arm**<br><br>♦ **HbA1c**<br>3 mo AR ↓0.52% (↓6.6%)<br>12mo AR ↓0.23% (↓2.9%)<br>Time effect SS<br><br>♦ **Weight**<br>3 mo AR ↓2.79kg (↓2.9%)<br>12mo AR ↓2.23kg (↓2.4%)<br>Time effect SS<br><br>♦ **HDL**<br>12 mo AI ↑0.08mmol/L (↑6.7%)<br>Time effect SS<br><br>♦**TRG** 12 mo AR ↓0.47mmol/L (↓19.7%)<br>Time effect SS<br><br>♦ **LDL** WG nSS	**Does not support claims**
**9**	Sato J, 2017[94]	RCT	To compared low carbohydrate diet (130gr) with calorie restricted diet in poorly controlled T2D in Japan	n=66 adults with T2D, 49 (94%) completed 6-month follow-up	6 months	**BG results favored low-carb.**<br><br>♦ **HbA1c** AR↓0.65% WG SS, BG SS<br><br>♦ **Weight** AR↓1.6kg WG SS, BG SS<br><br>♦ **TRG, LDL, HDL** BG nSS	**Support claims**
**Crossover trials**							
**10**	Boden G, 2005[95]	Crossover trial	Metabolic ward study.Compared effects first 1-week of a usual care diet (hospital food + food from "outside") followed by strict low-carb diet for 2 weeks.	n=10 obese adults with T2D<br><br>10 (100%) completed study.	7 days on usual care diet; 14 days on low-carb diet	**Results after 2 weeks carbohydrate restriction:**<br><br>♦ **HbA1c** AR ↓0.5% (↓6.8%) SS<br><br>♦ **FBG** AR ↓22.0mg/dL (↓16.3%) SS<br><br>♦ **LDL-C** WG nSS<br><br>♦ **Weight loss after subtraction of water weight loss** AR ↓1.65kg (↓2.4%) SS	**Supports claims.**

						♦ **Insulin sensitivity** Serum insulin ↓ SS<br><br>♦ **Rate of insulin-stimulated glucose disappearance** ↑200% SS<br><br>♦ **TRG** AR ↓57.5mg/dL (↓35.3%) SS<br><br>♦ **LDL, HDL** nSS<br><br>♦ **Total C** AR ↓17mg/dL (↓9.4%) SS	
**11**	Gannon MC, 2004[96]	Crossover RCT	Investigated the effect on glycemic control of a non-ketogenic low-carb, high-protein diet in adults with T2D. The test diet was the formulated low-biologically-available-glucose (LoBAG) diet. Weight loss was not a goal of this study. Control diet was based on recommendations of the American Heart Association.	n=8 overweight men with T2D<br><br>8 (100%) completed study.	5 weeks on each diet with a 5-week washout between the two diets	**For the low carb diet:**<br>♦ **FBG** AR ↓48mg/dL (↓28.7%) SS<br><br>♦ **Fasting insulin** nSS<br><br>♦ **Mean 24-h integrated net glucose area response** ↓77% SS<br><br>♦ **Total 24-h integrated glucose area response** ↓36.3% SS<br><br>♦ **TRG** AR ↓20mg/dL (↓8.8%) SS<br><br>♦ **Total C, HDL, LDL** BG and WG nSS	**Supports claims.**
**Non-randomized trials**							
**12**	Haimoto H, 2008 [97]	RCT	Compared low-carb diet vs. conventional diet. Results at 1 and 2 years.	n=133 adults with T2D<br><br>102 (77%) completed study.	2 years	**BG SS findings favored low-carb.**<br><br>♦ **HbA1c** AR ↓0.7% (↓9.5%) BG SS<br><br>♦ **BMI** AR ↓1.3kg/m^2 (↓5.2%) BG SS<br><br>♦ **LDL** AR ↓5mg/dL (↓4.1%) BG SS<br><br>♦ **Total C** AR ↓5mg/dL (↓2.4%) BG SS<br><br>♦ **Diabetes medication use**<br># patients on sulfonylureas reduced 43%, 57%, 94% for 3 drug types.<br>.	**Partially supports claims.**
**13**	Sanada M, 2018[98]	Single arm retrospective	Sought to determine if a moderately low-carb intervention is safe and	n=200 adults with T2D	36 months	♦ **HbA1c** AR ↓.5% (↓6.3%) SS<br><br>♦ **Total C** AR ↓6.7mg/dL (↓3.3%) SS	**Supports claims.**

			efficacious as determined by safety data and HbA1c, lipids, blood pressure, liver and renal function and weight over 36 months in a Japanese population.	157 (79%) completed 36-month follow-up.		♦ **LDL** AR ↓8.9mg/dL (↓7.7%) SS<br><br>♦ **TRG** nSS<br><br>♦ **DBP AR** SS; **SBP** nSS<br><br>♦ **Overall insulin and SU decreased and metformin and DPP-4 increased over 36 months**<br><br>♦ **Fasting glucose, HDL, TRG, weight** nSS	
**14**	Hallberg S, 2018 [99]	Non-random-ized, controlled parallel-arm trial	Outcomes (see McKenzie 2017) for ongoing trial assessing the effectiveness and safety of a remote, continuous care intervention combined with a very low-carb ketogenic diet for T2D management. Compared to usual care.	n=349 overweight and obese adults with T2D<br><br>218 (83%) in test diet group completed.	1 year	**The BG SS findings favored low-carb.**<br><br>♦ **HbA1c** AR ↓1.3% (↓17.1%) BG SS<br><br>♦ **FBG** AR ↓1.95mmol/L (↓21.9%) BG SS<br><br>♦ **Weight** AR ↓13.8kg (↓11.8%) BG SS<br><br>♦ **HOMA-IR** AR ↓6.82 (↓55%) BG SS<br><br>♦ **TRG** AR ↓0.54mmol/L (↓24.2%) BG SS<br><br>♦ **HDL** AI ↑0.20mmol/L (↑18.3%) BG SS<br><br>♦ **LDL** AI ↑0.26mmol/L (↑.3%) BG SS<br><br>♦ **Total C:HDL ratio** AR ↓0.53 (10.8%) BG SS<br><br>♦ **SBP** AR ↓6.36mmHg (↓4.8%) BG SS<br><br>♦ **DBP** AR ↓3.51mmHg (↓4.3%) BG SS<br><br>♦ **Diabetes medication use** Insulin therapy was reduced or eliminated in 94% of users; sulfonylureas were entirely	**Supports claims.**

						eliminated. Medications increased in the control group.<br><br>♦ **No adverse events** attributed to intervention.	
**15**	Krebs J, 2013 [100]	Single-arm trial	Tested intervention based on the Atkins diet; with 3 phases and gradual increase of carb intake over time.	**n=**14 obese adults with T2D<br><br>12 (86%) completed study.	24 weeks	♦ **HbA1c** At week 24 AR ↓1.1% (↓14.9%) SS<br><br>♦ **FBG** At week 12 AR ↓2.1mmol/L (↓21.6%) SS; at week 24 AR ↓1.7mmol/L (↓17.5%) nSS<br><br>♦ **Weight** At week 24 AR ↓9.7kg (↓8.1%) SS<br><br>♦ **HOMA** At week 12 SS improved; at week 24 nSS<br><br>♦ **HDL** At week 24 AI ↑0.14mmol/L (↑17.5%) SS<br><br>♦ **LDL** AI ↑0.40mmol/L (↑12.1%) SS<br><br>♦ **TRG** AR ↓0.37mmol/L (↓25.0%) nSS<br><br>♦ **SBP, DBP** nSS	**Supports claims.**
**16**	Hussain T, 2012 [101]	Non-random-ized, 2-arm trial	Compared very low carb, ketogenic diet vs. E-restricted diet. Participants allowed to select diet.	n=363 overweight and obese adults; 102 had T2D<br><br>102 of those with T2D (100%) completed study.	24 weeks	**For low carbohydrate:**<br><br>♦ **HbA1c** AR ↓1.2% (↓~19.2%) BG SS<br><br>♦ **Weight** AR ↓12.5kg (↓12.0%) BG SS<br><br>♦ **HDL, total C, TRG, LDL** SS improved<br><br>♦ **Adverse effects**. Urea levels increased SS. The uric acid and creatinine levels decreased.	**Supports claims.**
**17**	Sasakabe T, 2011 [102]	Single-arm trial	Investigated impacts on CVD risk and abdominal fat of a moderately	n=63 overweight and obese adults with T2D	6 months	♦ **HbA1c**<br>**Men** AR ↓1.9% (↓22.6%) SS<br>**Women** AR ↓1.6% (↓18.6%) SS	**Supports claims.**

			low-carb diet in adults with T2D.<br>Two arms were given different instructions based on participants' HbA1c levels. Those below <9.0% were asked to eliminate carb-rich foods from their dinner; patients with an HbA1c level ≥ 9.0% were asked to eliminate carbs from breakfast and dinner.	52 (83%) completed study.		♦ **FBG**<br>**Men** AR ↓21mg/dL (↓13.4%) SS<br>**Women** AR ↓20mg/dL (↓12.2%) nSS<br><br>♦ **Weight**<br>**Men AR** ↓2kg (↓2.8%) SS<br>**Women AR** ↓1.7kg (↓3.0%) SS<br><br>♦ **Fasting insulin** nSS<br><br>♦ **TRG** nSS<br><br>♦ **LDL**<br>Men nSS<br>Women AR ↓24mg/dL (↓15.3%) SS<br><br>♦ **DBP, SBP** nSS<br><br>♦ Visceral and subcutaneous adipose tissue, waist size SS ↓ men and women	
**18**	Nielsen J, 2008 [103]	Follow-up to a non-randomized2-arm trial	Follow up to non-randomized controlled trial Nielsen 2005. Compared the effects on glycemic control and weight of a low-carb diet vs. a high-carb diet at 3, 6, 22, and 44 months.	n in analysis at 44 mo.=23 obese adults with T2D	Original trial was 6 months; follow-up at 44 months	**For low carbohydrate:**<br><br>♦ **HbA1c** AR ↓1.2% (↓15.0%) SS<br><br>♦ **Weight loss** AR ↓7.5kg (↓7.5%) SS<br><br>♦ **HDL AI** ↑0.2mmol/L (↑18.2%) SS<br><br>♦ **Total C, TRG, TG:HDL ratio** nSS; **LDL** not reported.<br><br>♦ **HDL:total C ratio** AI ↑.2 (↑18.2%) SS<br><br>♦ **Diabetes medication use**<br>Mean insulin use increase from18u at 6 months to 41u at 44 months. Start of study mean was 60u.	**Supports claims.**
**19**	Nielsen J, 2005 [104]	non-randomized controlled trial	Compared a low-carbohydrate to a low-fat diet both calorie restricted on fasting glucose,	Total N =31 with diabetes<br>16 - low carbohydrate<br>15 calorie restricted	6 months trial with 1-year	**6 months for low carbohydrate:**<br>♦ **HbA1c** AR ↓1.4% (↓18%)<br><br>♦ **Lipids** not reported	**Supports claims**

			HbA1c, weight and BMI. 6 month intervention for both groups and then 1 year follow up. Full data not available.	low fat	follow up in low carb arm only	♦ **Diabetes medication use**<br>Insulin reduction but not elimination in 8 of 11 insulin treated patients with average dose 60u reduced to18u.<br>Insulin eliminated in 3 patients.<br>SFU eliminated in 3 and reduced in 2<br><br>**1 year:**<br>♦ **HbA1c** AR ↓1.0% (↓13%)	
**20**	Dashti H, 2007[105]	non-random-ized trial	Compared effects of low-carb, ketogenic diet on obese adults with normal blood glucose and obese adults with T2D.	Total n=64; 31 had T2D<br><br>100% completed. T2D sub-analysis conducted.	56 weeks	♦ **FBG** AR ↓5.6mmol/l (↓53.5%) SS<br><br>♦ **Weight** AR ↓24.5kg (↓22.7%) SS<br><br>♦ **HDL** AI ↑0.55mmol/L (↑53.5%) SS<br><br>♦ **LDL** AR ↓1.78mmol/L (↓34.5%) SS<br><br>♦ **TRG** AR ↓3.68mmol/L (↓78.5%) SS<br><br>♦ **Adverse effects.** Urea decreased SS. "No significant alteration was noticed in renal function test."	**Supports claims.**
**21**	Yancy W, 2005[106]	single-arm trial	Tested the effectiveness of a low-carb, ketogenic diet for improving glycemic control in individuals with T2D	n=28 overweight and obese adults with T2D<br><br>21 (75%) completed the study.	16 weeks	♦ **HbA1c** AR ↓1.2% (↓16.0%) SS<br><br>♦ **FBG** AR ↓1.5mmol/L (↓16.6%) SS<br><br>♦ **Weight** AR ↓8.7kg (↓6.6%) SS<br><br>♦ **HDL** AI ↑0.07mmol/L (↑7.6%) SS<br><br>♦ **TRG** AR ↓1.12mmol/L (↓41.6%) SS<br><br>♦ **Total C**, **LDL** WG nSS<br><br>♦ **SBP, DBP** nSS<br><br>♦ **Diabetes medication use**<br>Reduced or discontinued for 81% of subjects.<br><br>♦ **Renal function** Urea, creatinine nSS	**Supports claims.**

						♦ **Adverse events.** None related to the diet.	
**22**	Dashti H, 2004[107]	single-arm trial	To determine effects of ketogenic diet on glucose, weight, and lipids	n=83 obese adults with high FBG (>7 mmol/L or 125mg/dL). Sample mean=7.26mmol/L; 131mg/dL.<br><br>n completing study unknown	24 weeks	♦ **FBG** AR↓ 1.6mmol/L (↓22.6%) SS<br><br>♦ **Weight** AR↓ 14.4kg (↓14.2%) SS<br><br>♦ **HDL** ↑ SS<br><br>♦ **Total C**, **LDL** ↓ SS<br><br>♦ **TRG** AR ↓1.7mmol/L (↓60.4%) SS<br><br>♦ **Renal function** urea and creatinine nSS	**Supports claims.**
**Systematic Reviews and Systematic Review/Meta-analyses**							
**23**	Huntriss R, 2017[108]	Systematic review and meta-analysis	Evaluated the effects on HbA1c and weight loss of a low-carb diet in T2D. Reviewed 18 RCTs; 7 studies in quantitative meta-analysis.	Pooled n=2204 adults with T2D<br><br>18 RCTs	3 months -4 years	**1-year outcomes:**<br><br>♦ **HBA1c** (10 studies) Low-carb ↓ BG SS in 4 studies. AR range ↓0.02-1.2%; -0.28% WMD. 1 BG SS for control.<br><br>♦ **Weight loss** (10 studies) ↓ Low-carb ↓ BG SS in 3 studies. AR range: ↓0.9kg- 7.5kg. 0 studies BG SS for control. Pooled analysis: BG nSS**.**<br><br>♦ **HDL** (7 studies) Low-carb ↑ BG SS in all studies. 0.06mmol/L WMD.<br><br>♦**TRG** (7 studies) Low-carb ↓ BG SS improved in all studies. -0.24mmol/L WMD..<br><br>♦ **LDL** (5 studies) BG nSS in all.<br><br>♦ **Total C** (7 studies) BG nSS in all.<br><br>♦ **SBP** (7 studies) Low-carb ↓ BG SS in 2 studies. -2.74mg/dL mean difference. No study favored control.	**Supports claims.**

						♦ **DBP** (7 studies) Low-carb ↓ BG SS in 2 studies. No study favored control. Pooled analysis: BG nSS.<br><br>♦ **Diabetes medication use**<br>In 9 of 14 studies with data, reduction was SS greater with low-carb (insulin, hypoglycemic agents or combined meds score).<br><br>♦ **Compliance**: Authors concluded that dietary adherence was a problem in most studies reviewed and that <50g carb/day was unrealistic and that <130g carb/day was more achievable.	
**24**	Ajala O, 2013[40]	Systematic review and meta-analysis	Assessed the effect of diet types on glycemic control, lipids, and weight loss. 20 RCTs included. 16 studies included in meta-analysis.<br><br>Compared low-carbohydrate; vegetarian; vegan; low-glycemic index (GI); high-fiber; Mediterranean; high-protein diets vs. control diets (low-fat; high-GI; ADA; European Association for the Study of Diabetes; and low-protein diets).	n in analyses= 3073 adults T2D<br><br>20 RCTs	6 mos-4 years	**Low-carb compared to other diets:**<br><br>♦ **HbA1c** ↓ BG SS -0.12% WMD<br><br>♦ **Weight** BG nSS<br><br>♦ **HDL** ↑ BG SS +0.08mmol/L WMD<br><br>♦ **TRG** BG nSS<br><br>♦ **LDL** BG nSS	**Partially supports claims.**
**25**	Castañeda-González LM, 2011[109]	Systematic review	Reviewed 8 trials (2000-2010) ≥ 12 weeks duration, to evaluate longer-term effects of low-carb diet compared to low-fat, low-carb Mediterranean diet, usual care diet, healthy eating diet, or low-glycemic index diet.	Adults with T2D<br><br>8 RCTs	Range: 3-48 months	♦ **HbA1c** 6 studies showed greater reduction with low-carb, 2 BG SS.<br><br>♦ **Weight** 5 studies showed greater loss with low-carb, 1 BG SS. The longest trial did not show a difference in weight change.<br><br>♦ **IR** Improved in 1 study.	**Limited support for claims.**

						♦ **Lipids** mixed results compared to control. Improved in several studies.	
**26**	Sainsbury E, 2018 [110]	Systematic review/meta-analysis	Assessed the effects of carb-restricted diets (≤45%E) compared to high-carb diets (>45%E) on glycemic control in adults with T2D.<br><br>25 studies.<br>Meta-analyses by diet protocol carb target level (low, moderate, high).	Pooled n=2412<br>24 studies on T2D; 1 study on T1D		♦ **HbA1c** (22 studies):<br>**WG changes:** AR ↓0.77% WG SS (all carb-restricted diets) vs AR ↓0.50% WG SS (high-carb diets)<br><br>**BG changes:**<br>at 3 mo, WMD -0.47% BG SS (favoring low-carb vs high-carb); BG nSS for mod-carb vs high carb.<br><br>at 6 mo, WMD -0.36% BG SS (favoring low-carb vs high-carb); BG nSS for mod-carb vs high carb.<br><br>at 12 and 24 mo, BG nSS<br><br>♦ **Weight**<br>at 3 mos, WMD -1.08kg (favoring all carb-restricted diets vs high-carb diets). BG SS. All improvement attributed to low-carb diets (AR ↓2.47 kg greater loss than high-carb).<br><br>at 6 mos, BG nSS<br><br>at 12 mos WMD -0.58kg (favoring mod-carb vs high-carb) BG SS<br><br>♦ **Diabetes medication use** (12 studies) Greater reduction on carb-restricted diets at all time points including insulin and oral medications.	**Supports claims.**
**27**	Schwingshackl L, 2018 [43]	Systemic review and network meta-analysis	Compared the efficacy of different dietary approaches on glycemic control in T2D. Nine dietary approaches were included low-fat, vegetarian, Mediterranean, high-protein,	Adults with T2D	≥ 12 weeks	♦ **HbA1c** AR↓0.82% vs control<br>♦ **Fasting glucose** AR↓1.23mmol/L vs control	**Supports claims.**

	moderate-carbohydrate, low-carbohydrate, control, low GI/GL, Paleolithic). Lit search through July 2017. 56 trials were included, published between 1978 and 2016.	

Abbreviations list for Supplementary tables 1-4:
T2D, type 2 diabetes; **BG**, between group; **WG**, within group; **FBG**, fasting blood glucose; **SS**, statistically significant; **nSS**, not statistically significant; **DBP**, diastolic blood pressure; **SBP**, systolic blood pressure; **HDL**, high-density lipoprotein; **TRG**, triglyceride; **DGA**, dietary guidelines for American; **RCT**, randomized controlled trial; **UC**, usual care; **low-carb**, low carbohydrate; **CHO**, carbohydrate; **Med**, Mediterranean; **LDL**, low-density lipoprotein; **BMI**, body mass index; **HOMA-IR**, homeostasis model assessment of insulin resistance; **CVD**, cardiovascular disease; **PBD**, plant based diet; **FU**, follow-up; **VLDL**, very low-density lipoprotein; **LFV**, low-fat vegan; **E**, energy; **GI**, glycemic index; **total C**, total cholesterol; **OB**, obese; **VLCK**, very low calorie ketogenic; **IL-IRa**, interleukin 1-receptor antagonist,: **IL-6**, interleukin-6

Supplemental Table S5 Standards for reviewing scientific evidence for clinical guidelines

System	Link	Year Published	Description	Summary	Focus
**GRADE**	http://gdt.guidelinedevelopment.org/app/handbook/handbook.html	2016	"Process of rating the quality of the best available evidence and developing health care recommendations following the approach proposed by the Grading of Recommendations, Assessment, Development and Evaluation (GRADE) Working Group"	"a transparent and structured process for developing and presenting evidence summaries and for carrying out the steps involved in developing recommendations"	Evidence based summaries
**AGREE II**	https://www.agreetrust.org/wp-content/uploads/2017/12/AGREE-II-Users-Manual-and-23-item-Instrument-2009-Update-2017.pdf		"1. Assess the quality of guidelines; 2. Provide a methodological strategy for the development of guidelines; 3. Inform what information and how information ought to be reported in guidelines."	"developed to address the issue of variability in guideline quality"	Guidelines
**Guidelines International Network**	http://annals.org/aim/fullarticle/1103747/guidelines-international-network-toward-international-standards-clinical-practice-guidelines		"key components address panel composition, decision-making process, conflicts of interest, guideline objective, development methods, evidence review, basis of recommendations, ratings of evidence and recommendations, guideline review, updating processes, and funding"	"proposed set of key components for guideline development."	Guidelines
**Institute of Medicine**	http://www.nationalacademies.org/hmd/Reports/2011/Clinical-Practice-Guidelines-We-Can-Trust.aspx	2013	"proposed standards cover a number of elements essential to developing sound practice guidelines, including transparency; conflict of interest; guideline development group composition; CPG–SR intersection; establishing evidence foundations for and strength of recommendations; articulation of recommendations; external review; and updating"	The product of study to "develop a set of standards for developing rigorous, trustworthy clinical practice guidelines."	Clinical Practice Guidelines

Supplemental Table S6 Assessment and recommendations for improvement of the ADA guidelines using the National Academies of Sciences, Engineering and Medicine's *Clinical Practice We Can Trust* evaluation method

NAM Standard	ADA Report	ADA Critique	Recommendations
**Standard 1 - Establishing Transparency**	"The ADA adheres to the . "	Adherence to National Academy of Medicine Standards not explicitly defined.	Provide a table to ensure National Academy of Medicine Standards 1-7 are adhered to.
**"The processes by which a CPG is developed and funded should be detailed explicitly and publicly accessible.**	"Appointment to the PPC is based on excellence in clinical practice and research."	Process not explicitly described. Unknown how the Committee is selected.	Explicitly describe the process of selection. Publish all nominations and selected committee members before guidelines are published.
	"The ADA funds development of the Standards of Care out of its general revenues and does not use industry support for this purpose."	Funding not explicitly stated. Unknown funding supporting guidelines development	Specify where "general funds" originated and how influence from industry support to ADA activities is minimized.
**"Strategies for managing potential COI range from exclusion of conflicted members from direct panel participation or restriction of roles, to formal or informal consultation, to participation in certain exclusive recommendations, to simple disclosure of COI."**	"All members of the PPC are required to disclose potential conflicts of interest with industry and/or other relevant organizations"	Most members of PPC were conflicted: Only 3/14 on SOC committee had no COI, and only 5/11 on Nutrition Therapy Panel had no COI	Describe how conflicts amongst members of the review are managed<br><br>Nam recommends that not more than a minority of members should have a COI so this standard should be upheld<br><br>Ensure that no funders have COI roles and make this information available
**Standard 2 - Management of conflict of interest**			
**2.1 Individuals being considered for membership should declare all interests and activities potentially resulting in COI**	"All members of the PPC are required to disclose potential conflicts of interest with industry and/or other relevant organizations. "	Patient and public not obviously involved	Consider COI for membership. State policy
**2.2 COI disclosure and discussion**	"Members of the committee, their employers, and their disclosed conflicts of interest are listed in the "Professional Practice Committee Disclosures" table"	COI not explicitly considered prior to membership composition	Patient representation from both type1 and type 2 community
**2.3 Divestment**	None	Divestment policy not stated	Whenever possible guideline development group members should not have COI
**2.4 Exclusions**	None	Exclusion policy not stated	
**Standard 3 - Guideline development group composition**			Compose multidisciplinary & balanced guideline development group, with each component explicitly defined
**3.1 Multidisciplinary & Balanced**	"The PPC is a multidisciplinary expert committee comprised of physicians, diabetes educators, registered dietitians, and others	Not uniformly applied	Ensure patient (Type 1 and Type 2)and public involvement by recruiting additional members to the guideline development group.

	who have expertise in a range of areas, including adult and pediatric endocrinology, epidemiology, public health, lipid research, hypertension, preconception planning, and pregnancy care.”		
**3.2 Patient & Public Involvement**	Unknown	None	"Selection criteria should be applied to choose a consumer representative who can consider the evidence objectively, and make recommendations departing from preconceived views of self or interests" ADA might invite patients or other laypersons to review draft documents or attend a meeting to share perspectives.
**3.3 Strategies to increase participation by patient & public**			
**Standard 4 - Clinical Practice Guideline–Systematic Review Intersection**			
**4.1. Use systematic reviews that meet IOM standards**	Unknown	Unknown	
**4.1. Systematic reviews should coordinate with Guideline Development Review Team**	"PPC members systematically searched MEDLINE for human studies related to each section"	No search criteria identified	
**5. Establishing Evidence Foundations for and Rating Strength of Recommendations**			
**5.1. Components: For each recommendation, the following should be provided**			
**-- Underlying reasoning**	Variable	Not uniformly applied	Define harms/benefits
**-- Potential Harms/Benefits**	Variable	Not uniformly applied	Define relevant available evidence
**-- Summarize Relevant Available Evidence**	Variable	Not uniformly applied	Define quality
**-- Description of Quality**	Variable	Not uniformly applied	Define quantity and consistency
**-- Description of Quantity and consistency**	Variable	Not uniformly applied	Define part played by values, opinion, theory, and clinical experience
**-- Explanation of the part played by values, opinion, theory, and clinical experience in deriving the recommendation.**	None	Not performed	Uniformly apply rating to level of confidence
**-- A rating of the level of confidence in (certainty regarding) the evidence underpinning the recommendation**	Variable	Not uniformly applied	
**6. Articulation of Recommendations**			Implement standard reporting tools
**6.1 Standard reporting: Recommendations should be articulated in a standardized form detailing precisely what the**	None	Form not standardized	Define recommendations precisely

**recommended action is, and under what circumstances it should be performed**			
**6.2 Precise recommendations: Strong recommendations should be worded so that compliance with the recommendation(s) can be evaluated.**	None	Recommendations not clearly articulated	Implement formal external review
**7. External Review**	Unknown. A process to submit comments has been established (“Readers who wish to comment on the 2018 Standards of Medical Care in Diabetes are encouraged to do so. All suggestions will be reviewed by the Association and the Professional Practice Committee.”)	External review not stated	Recruit external reviewers with a full diversity of experiences
**7.1 Diversity of experiences: External reviewers should comprise a full spectrum of relevant stakeholders,**	None		Develop systems to ensure confidentiality of external reviews
**7.2 Confidentiality: The authorship of external reviews submitted by individuals and/or organizations should be kept confidential**	None	Confidentiality of comments is not explicitly assured	

TABLE S7 Summary of evaluation of studies on different eating patterns cited in the American Diabetes Association 2018 guidelines and 2014 nutrition recommendations

Study #	ADA statement/recommendation	Citation: first author, year	Study type	Description	Participants	Duration	Findings for test diet	Comments
DASH DIET								
1	"A variety of eating patterns (combinations of different foods or food groups) are acceptable for the management of diabetes. In one small study in people with type 2 diabetes, the DASH eating plan improved A1c, blood pressure, and other cardiovascular risk factors." (46, ADA 2014)	Azadbakht L, 2011 [ref. 46, ADA 2014][15]	Randomized crossover	Compared DASH diet to control diet. Calorie and macronutrient distribution same in both	n = 44 persons with T2D<br>31 (70%) completed study	8 wk each diet	◆ **HbA1c** AR ↓1.7% (↓22.1%) BG SS<br>◆ **FBG**<br>AR ↓29.4 mg/dL (↓18.3%) BG SS<br>◆ **Weight** AR ↓5.0 kg (↓6.8%) BG SS<br>◆ **HDL** AI ↑4.3 mg/dL (↑10.4%) BG SS<br>◆ **SBP** AR ↓13.6 mm Hg (↓10.1%); **DBP** AR ↓9.5 mm Hg (↓11.6%) BG SS<br>◆ **TRG** AI ↑14.4 mg/dL (↑8.4%) BG SS unknown	**Should be included in a review of the evidence**<br>Supports the ADA statement<br>Error in reporting and analysis of triglycerides
2	"The blood pressure benefits are thought to be due to the total eating pattern, including the reduction in sodium and other foods and nutrients that have been shown to influence blood pressure." (99, 105, ADA 2014)	Harsha DW, 1999 [ref. 99, ADA 2014][16]	RCT	Multicentre DASH trial. Compared control diet (typical fat content for US population); diet rich in fruit and vegetables; DASH (fruit, vegetables, low fat)	n = 459 persons with SBP <160 mm Hg, DBP 80 to 95 mm Hg<br>354 (77%) completed study	8 wk	No data on persons with diabetes<br>**Differences between DASH and control:**<br>◆ **SBP** ↓ 5.5 mm Hg BG SS<br>◆ **DBP** ↓3.0 mm Hg BG SS	**Should not be included in review of evidence**<br>This study excluded subjects with T2D
3		US HHS, USDA Dietary Guidelines for Americans, 2010 [ref. 105, ADA 2014][17]	Health policy report	Diet recommendations for the general population; includes sodium limit for blood pressure control				**Should not be included in a review of the evidence**<br>DGAs are not intended for persons with diabetes.
4	"In people without diabetes, the DASH eating plan has been shown to help control blood pressure and lower risk for CVD and is frequently recommended as a healthful eating pattern for the general population (104-106, ADA,	Sacks FM, 2001 [ref. 104, ADA 2014][18]	RCT	Compared DASH and control diets (typical US diet). Within each group, participants ate foods with high, mid, and low sodium for 30 days each, in random order	n = 412 persons with SBP 120 to 159 mm Hg, DBP 80 to 95 mm Hg<br>390 (95%) completed study	8 wk	**Differences between DASH and control for 3 Na levels:**<br>◆ High Na-intake group: **SBP** ↓5.9 mm Hg; **DBP** AR↓ 2.9 BG SS<br>◆ Mid Na-intake group: **SBP** ↓5.0 mm Hg; **DBP** ↓ 2.5 mm Hg BG SS	**Should not be included in a review of the evidence**<br>Study does not include separate analysis of subjects with T2D

TABLE S7 (Continued)

Study #	ADA statement/recommendation	Citation: first author, year	Study type	Description	Participants	Duration	Findings for test diet	Comments
	2014). Limited evidence exists on the effects of the DASH eating plan on health outcomes specifically in individuals with diabetes; however, one would expect similar results to other studies using the DASH eating plan." (46, ADA 2014)						◆ Low Na-intake group: **SBP** ↓2.2 mm Hg; **DBP** ↓1.0 mm Hg BG SS	
5		Appel L, 1997 [ref. 106, ADA 2014][19]	RCT	Compared effects of three dietary patterns (control, typical diet; diet rich in fruits and vegetables; in DASH [fruit + veg., low-fat]). Sodium levels same for all diets	n = 459 persons with SBP <160 mm Hg; DBP 80 to 95 mm Hg<br>~97% across groups completed study	8 wk	No data on persons with diabetes<br>**Differences between DASH and control:**<br>• **SBP** ↓5.5 mm Hg BG SS<br>**DBP** ↓3.0 mm Hg BG SS	**Should not be included in review of evidence**<br>Study does not include analysis of subjects with T2D
6	The DASH diet is an example "of healthful eating patterns that have shown positive results in research." (56-58, ADA 2018)	Cespedes EM, 2016 [ref. 58, ADA 2018][20]	Observational data analysis from an RCT (Women's Health Initiative)	Investigated incidence of T2D in adherents of four dietary patterns including DASH	n = 101 504 postmenopausal women without T2D		◆ Lowest incidence of T2D associated with adherence to DASH	**Should not be included in review of evidence**<br>This may belong in the prevention section but not appropriate in the management section
7		Ley SH, 2014 [ref. 59, ADA 2018][21]	Non-systematic review	Cites two prospective studies on DASH on food intake and T2D incidence for diabetes prevention and Azadbakht, 2011 trial on diabetes management			◆ DASH associated with lower T2D risk	**Should not be included in review of evidence**<br>It is not a systematic review
8		Campbell AP, 2017 [ref. 56, ADA 2018][22]	Commentary	Presents basics of DASH diet. Cites one RCT on DASH in persons with T2D				**Should not be included in a review of the evidence**<br>This was a commentary not based on a systematic review. Cites only one study on DASH in persons with T2D

TABLE S7 (Continued)

Study #	ADA statement/recommendation	Citation: first author, year	Study type	Description	Participants	Duration	Findings for test diet	Comments
MEDITERRANEAN DIET								
1	"The Mediterranean-style eating pattern … has been observed to improve cardiovascular risk factors (ie, lipids, blood pressure, triglycerides) in individuals with diabetes… (11, 72, 88, 100, ADA 2014) Individuals following an energy-restricted Mediterranean-style eating pattern also achieve improvements in glycaemic control." (88, ADA 2014).	Esposito, 2009 [ref. 72, ADA 2014; ref. 54, ADA 2018][25]	RCT	Tests diet for efficacy in delay of medication initiation in patients newly diagnosed with T2D.<br>Mediterranean low-carbohydrate diet (<50% E CHO) vs. control diet (<30%E fat). Both diets were E-restricted	n = 215 overweight persons newly diagnosed with T2D<br>195 (91%) completed study	4 y	◆ **HbA1c** y 4: AR ↓0.9 (↓11.6%) **BG SS**<br>◆ **Weight** superior to control, **BG SS** at y 1 but nSS at y 4<br>◆ **TRG** year 4: AR ↓0.28 mmol/L (↓14.7%) **BG SS**<br>◆ **HDL** year 4: AI ↑0.09 mmol/L (↑8.2%) **BG SS**<br>◆ **% requiring meds at year 4:** Mediterranean diet: 44%<br>Control: 70% BG SS	**Should be included in a review of the evidence**<br>Supports the ADA statement with limitations: Some benefits not sustained. 44% required medication by end of study
2	"The Mediterranean-style eating pattern … has been observed to lower combined endpoints for CVD events and stroke when supplemented with mixed nuts … or olive oil." (83, ADA 2014).	Estruch, 2013 [ref. 83 ADA 2014][26] replaced by Estruch 2018 after re-analyses[27]	RCT PREDIMED study	CV events was main outcome of interest<br>Compared two versions of Mediterranean diet to low-fat control diet. Not E-restricted	n = 3614 persons with T2D	4.8 y	◆ The two Mediterranean diets reduced the incidence of major CVD events (composite of myocardial infarction, stroke and death from cardiovascular causes)<br>◆ **Overall study population** Mediterranean diet with olive oil: HR = 0.69<br>Mediterranean diet with nuts: HR = 0.74	**Should be included in a review of the evidence**<br>Supports the ADA statement in regard to CVD risk
3		Elhayany, 2010 [ref. 100, ADA 2014][28]	RCT	Compared low-carbohydrate Mediterranean diet, traditional Mediterranean diet, and 2003 ADA diet. Limited to 20 cal/kg body weight in all study groups<br>Main outcomes: HbA1c, FBG, TRG	n = 259 persons with T2D<br>194 (75%) completed study	12mo	◆ **HbA1c** low-carbohydrate Mediterranean AR ↓2.0% (↓24.1%) BG SS (vs. ADA diet); traditional Mediterranean AR ↓1.8% (↓21.7%) BG SS (vs ADA diet)<br>◆ **weight loss** low-carbohydrate Mediterranean AR↓8.9 kg (↓10.3%) BG nSS<br>Traditional Mediterranean AR ↓7.4 kg (↓8.7%) BG nSS but WG SS<br>◆ **HDL** low-carbohydrate Mediterranean AI↑	**Should be included in a review of the evidence**<br>Supports the ADA statement

TABLE S7 (Continued)

Study #	ADA statement/recommendation	Citation: first author, year	Study type	Description	Participants	Duration	Findings for test diet	Comments
							0.13 mmol/L (↑12.0%) BG SS<br>(vs ADA and Traditional Mediterranean diets)<br>◆ **TRG** low-carbohydrate Mediterranean AR<br>↓1.52 mmol/L (↓47.8%) BG SS (vs ADA diet); traditional Mediterranean AR↓1.46 mmol/L (↓48.0%) BG SS (vs ADA diet)<br>◆ **LDL** low-carbohydrate Mediterranean AR<br>↓.61 mmol/L (↓20.0%) BG SS (vs ADA diet); traditional Mediterranean AR↓<br>.55 mmol/L (↓17.3%) BG nSS	
4		Wheeler, 2013 [ref. 88, ADA 2014][29]	Systematic review	2001 to 2010 review on diet and diabetes; cites seven studies on Mediterranean diet		4 wk to 4 y	"There appears to be no advantage in using the Mediterranean-style eating pattern compared with other eating patterns for glycaemic control. There are mixed results for CVD risk factors with some studies indicating<br>that the Mediterranean-style eating pattern might improve HDL cholesterol and TG."	**Should be included in a review of the evidence**<br>Does not strongly support the ADA statement
5		Franz, 2010 [ref. 11, ADA 2014][30]	Systematic review	Reviews the evidence for the ADA's nutrition practice guidelines. In section on treatment for CVD, cited are studies on Mediterranean diet that include two RCTS, two cross-sectional and one case-control study	Persons with diabetes or other CVD risk factors		Notes that the Mediterranean diet has shown benefit for endothelial health, blood pressure and lipid levels, but concludes that "a clearer understanding is needed" of the diet's "protective mechanisms and role in diabetes management."	**Should be included in a review of the evidence**<br>Does not strongly support the ADA statement

TABLE S7 (Continued)

Study #	ADA statement/recommendation	Citation: first author, year	Study type	Description	Participants	Duration	Findings for test diet	Comments
6	"A variety of eating patterns are acceptable for persons with diabetes." The Mediterranean diet is an example. (54 Esposito, above; 55, ADA 2018)	Boucher, 2018 [ref. 55, ADA 2018][31]	Commentary based on non-systematic review	Discussions of select studies on the Mediterranean diet in diabetes				**Should not be included in a review of the evidence**<br>This was a commentary that was not based on a systematic review.
PLANT-BASED DIET								
1	"A variety of eating patterns are acceptable for the management of diabetes." Plant-based diets are an example of a healthy eating pattern. (59, 60, ADA 2018)	Rinaldi S, 2015 [ref. 57, ADA 2018][47]	A non-systematic review	Literature search through March 2015. Reviewed 13 studies (five RCTs, four observational, three follow-up or ancillary to cited RCTs, one meta-analysis) on glycaemic control, CVD risk, other health measures			Results mixed on efficacy of PBD for glycaemic control, weight loss, CVD risk improvement	**Should not be included in a review of the evidence**<br>Not a systematic review<br>Limitations: Of five RCTs, two had n < 20, short duration (4, 12 weeks)
2		Pawlak R, 2017 [ref. 60, ADA 2018][48]	Commentary based on non-systematic review	Reviews observational studies on diabetes				**Should not be included in a review of the evidence**<br>This was a commentary not based on a systematic review
3	Six plant-based diets reviewed found inconsistent results for glycaemic control and weight loss. "Diets often did result in weight loss." (36, 93, 101-103 131, ADA 2014).	Barnard N, 2006 [ref. 36, ADA 2014][49]	RCT	Compared low-fat vegan diet to ADA diet for glycaemic control and CVD risk. Only the control diet was E-restricted	n = 99 persons with T2D 88 (88%) completed study	22 wk	**For vegan diet:**<br>◆ **HbA1c** AR ↓0.96% (↓12.0%) WG SS; BG nSS<br>◆ **FBG** AR ↓35.5 mg/dL (↓21.7%) WG SS, BG nSS<br>◆ **Weight** AR ↓5.8 kg (↓6.0%) WG SS, BG nSS<br>◆ **Diabetic meds** ↓43% **BG SS**<br>◆ **HDL**↑ **LDL**↓ **VLDL**↓ **TRG**↓<br>All WG SS. All BG nSS	**Should be included in a review of the evidence**<br>Supports the ADA statement
4		Turner-McGrievy, 2008 [ref. 93, ADA 2014][50]	Nutritional assessment of diets tested in Barnard, 2006 trial	Ancillary study to 22-wk. trial (Barnard, 2006). Assessed changes from baseline of nutrient intake and diet quality of participants in vegan diet group vs. ADA diet, using AHEI metric based on the US DGA			The vegan diet group increased intakes of carbohydrate, fibre, some micronutrients and improved its AHEI score; the ADA diet group AHEI score stayed same	**Should not be included in a review of the evidence**<br>This study is not germane to glycaemic control or weight loss (see ADA statement)

TABLE S7 (Continued)

Study #	ADA statement/recommendation	Citation: first author, year	Study type	Description	Participants	Duration	Findings for test diet	Comments
5		Nicholson A, 1999 [ref. 101, ADA, 2014][51]	RCT	Compared non-isocaloric low-fat vegan and low-fat diets on glycaemic control and CVD risk factors. low-fat vegan prepared meals lower in E than those for low-fat meals risk.	n = 13 with T2D<br>11 (85%) completed study	12 wk	**For the vegan diet:**<br>◆ **HbA1c** AR↓1.4% (↓16.9%) BG nSS<br>◆ **FBG** AR↓ 54.0 mg/dL (↓27.8%) BG SS<br>◆ **weight** AR↓ 7.2 kg (7.4%) BG SS<br>◆ **lipids** all nSS	**Should be included in a review of the evidence**<br>Supports the ADA statement<br>Note: 11 completed study (control group n = 4)
6		Tonstad S, 2009 [ref. 102, ADA 2009] [52]	Cross-sectional	Assessed prevalence of T2D in the Adventist health study cohort for different types of vegetarian diets and in non-vegetarians	1007 in subgroup analysis of health measures		◆ **T2D prevalence** vegans 2.9%, non-vegetarians 7.6%<br>◆ **diabetes risk (OR)** vegans .51 (vs non-vegetarians, 1.0)<br>◆ **BMI** vegans 23.6 (lowest of all diet types) non-vegetarians 28.8	**Should not be included in a review of the evidence**<br>Observational studies cannot provide evidence for treatment efficacy<br>This study is not germane to glycaemic control or weight loss (see ADA statement)
7		Kahleova H, 2011 [ref. 103, ADA 2014] [53]	RCT	Compared E-restricted vegetarian and conventional diabetic diets on body fat, HbA1c oxidative stress. No exercise for first 12 weeks and then exercise added in second 12 weeks	n = 74 persons with T2D 62 (84%) completed study	24 wk	**For vegetarian diet:**<br>◆ **HbA1c** AR ↓0.65% (↓8.6%) WG SS BG nSS<br>◆ **weight** AR ↓6.2 kg (↓6.1%) BG SS<br>◆ **medication** ↓43% BG SS<br>◆ **LDL** AR ↓0.17 mmol/L (↓6.7%) WG SS, BG nSS<br>**Oxidative stress markers** improved SS more with vegetarian diet. ◆ **HDL, TRG** BG nSS	**Should be included in a review of the evidence**<br>Supports the ADA statement
8		Barnard N, 2009 [ref. 131, ADA, 2013] [54]	Follow-up to RCT Barnard 2006	Compared low-fat vegan and ADA diets for glycaemic control and CVD risk. 74-week follow-up to 22-week RCT (Barnard, 2006)	n = 99 with T2D<br>87 (88%) completed study	74 wk	**For vegan diet:**<br>◆ **HbA1c** AR ↓0.34% (↓4.2%) WG, BG nSS<br>◆ **Weight** AR ↓4.4 kg (↓4.5%) WG SS, BG nSS<br>**LDL**↓ **VLDL**↓ **TRG**↓ all WG SS in low-fat vegan, BG nSS<br>◆ **HbA1c before medication adjustment AR** ↓0.40% (before any medication changes) WG, BG SS	**Should be included in a review of the evidence**<br>Support for the ADA statement is limited

TABLE S7 (Continued)

Study #	ADA statement/recommendation	Citation: first author, year	Study type	Description	Participants	Duration	Findings for test diet	Comments
LOW-CARBOHYDRATE STUDIES								
1	1." Some published studies comparing lower levels of carbohydrate intake (ranging from 21 g daily up to 40% daily energy intake) to higher carbohydrate intake levels indicated improved markers of glycaemic control and insulin sensitivity with lower carbohydrate intakes." (92, 100, 107-111, ADA 2014)<br>2. "Many of these studies were small, were of short duration, and/or had low retention rates." (92 107, 109 110 112 113, ADA 2014)<br>3. "Some studies comparing lower levels of carbohydrate intake to higher carbohydrate intake levels revealed improvements in serum lipid/lipoprotein measures, including improved triglycerides, VLDL triglyceride, and VLDL cholesterol, total cholesterol, and HDL cholesterol levels (71,92 100 107 109 111, 112, 115)."<br>4. "Four RCTs indicated no significant difference in glycaemic markers with a lower-carbohydrate diet compared with higher carbohydrate intake	Stern L, 2004 [ref. 92, ADA 2014][69]	Follow-up to RCT Samaha 2003	1-y follow-up to 6-mo RCT in which patients followed either low-carbohydrate diet or E-restricted low-fat diet.	n = 54 obese persons with T2D (subgroup of larger study cohort)<br>34 (63%) of T2D subgroup completed study	1 y	◆ **For low-carbohydrate diet:** **HBA1c** AR ↓0.7% (↓10.8%) BG SS after adjustment and remained SS after weight loss was added to model<br>◆ **FBG** AR↓1.55 mmol/L (↓16.8%) BG nSS<br>◆ **insulin sensitivity** BG nSS<br>◆ lipid levels and weight change not reported for T2D subgroup	**Should be included in a review of the evidence**<br>Supports ADA statement 1, but not statement 3 (data not available for T2D)<br>Partial support for ADA statement 2. Note 1-y duration

TABLE S7 (Continued)

Study #	ADA statement/recommendation	Citation: first author, year	Study type	Description	Participants	Duration	Findings for test diet	Comments
	levels." (71, 112–114, ADA 2014)."<br>5. "A few studies found no significant difference in lipids and lipoproteins with a lower-carbohydrate diet compared with higher carbohydrate intake levels. It should be noted that these studies had low retention rates, which may lead to loss of statistical power and biased results (110 113 116, ADA)."							
2		Elhayany A, 2010 [ref. 100 in ADA 2014][28]	RCT	Compared low-carbohydrate Mediterranean diet, traditional Mediterranean diet, and 2003 ADA diet. All diets were E-restricted. All diets restricted calories to 20/kg. Of body weight	n = 259 overweight or obese persons with T2D 194 (75% completed study)	1 y	◆ **HbA1c** low-carbohydrate Mediterranean AR ↓2.0% (↓24.1%) BG SS (vs ADA diet)<br>◆ **weight loss** low-carbohydrate Mediterranean AR↓10.1 kg (↓10.3%) WG SS, BG nSS<br>◆ **HDL** low-carbohydrate Mediterranean AI↑ 0.13 mmol/L (↑12.0%) BG SS<br>With (vs ADA and traditional Mediterranean diets)<br>◆ **TRG** low-carbohydrate Mediterranean AR ↓1.52 mmol/L (↓47.8%) BG SS with (vs ADA diet)<br>◆ **LDL** low-carbohydrate Mediterranean AR ↓.61 mmol/L (↓20.0%) BG SS (vs ADA diet)	**Should be included in a review of the evidence**<br>Supports ADA statements 1 and 3
3		Miyashita Y, 2004 [ref. 107, ADA 2014][70]	RCT	Compared effects of isocaloric low-carbohydrate diet vs. high-carbohydrate diet, on glucose and lipid	n = 22 obese adults with T2D; n completed unknown	4 wk	**For low-carbohydrate diet:**<br>◆ **Fasting insulin** (↓30%) BG SS<br>◆ **FBG** AR ↓103 mg/dL (↓50.0%) BG nSS<br>◆ **HDL** ↑15% BG SS	**Should be included in a review of the evidence**<br>Supports ADA statements 1, 2, and 3

TABLE S7 (Continued)

Study #	ADA statement/recommendation	Citation: first author, year	Study type	Description	Participants	Duration	Findings for test diet	Comments
				metabolism, and visceral fat accumulation			◆ **TRG** BG nSS<br>◆ **FBG** AR ↓98 mg/dL ↓50.0% BG nSS<br>◆ **Weight loss** AR ↓9 kg ↓12.3% BG nSS<br>◆ **Visceral fat accumulation** BG SS favoured low-carbohydrate −40 cm (2) versus −10 cm(2)	Limitations: Carbohydrate intake unknown; n completed unknown
4		Shai I, 2008 [ref. 108, ADA 2014][36]	RCT	Compared safety and effectiveness of 3 diets (low-fat, restricted-E; Mediterranean, restricted-E; low-carbohydrate, not restricted-E) in obese persons, some with T2D.	322 obese adults, n with T2D = 46 (14%) for 36 included in analysis (low-carbohydrate n = 12, Mediterranean n = 13, low-fat n = 11).	2 y	◆ **HbA1c**: Low-fat decrease 0.4<br>Mediterranean decrease 0.5% low-carbohydrate AR↓ 0.9% WG SS only for low-carbohydrate, BG nSS<br>◆ **FBG, HOMA-IR, fasting insulin** nSS for low-carbohydrate	**Should be included in a review of the evidence.**<br>Supports ADA statement 1
5		Jonsson T, 2009 [ref. 109, ADA 2014][71]	RCT crossover	Compared Paleolithic diet vs. ADA diabetes diet for improving CVD risk.	**n = 17** adults with T2D; 13 (76%) completed study	3 mo on each diet	**Differences between paleo and ADA:**<br>◆ **HbA1c** ↓0.4% BG SS<br>◆ **TRG** ↓0.4 mmol/L BG SS<br>◆ **LDL** BG nSS<br>◆ **DBP** ↓4 mm Hg BG SS<br>◆ **HDL** ↑0.08 mmol/L BG SS<br>◆ **weight** ↓3 kg BG SS	**Should be included in a review of the evidence**<br>Supports ADA statements 1, 2, and 3
6		Khoo J, 2011 [ref. 110, ADA 2014][72]	RCT	Compared effects of two diets (low-calorie vs. low-calorie/high protein/low-fat) on weight loss, sexual and endothelial function, lower urinary track symptoms, and inflammatory markers in obese men	31 obese men with T2D n completed unknown	8 wk	**See comments**	**Should not be included in a review of the evidence**<br>Not relevant: Compares two restricted-E diets. No evidence that the test diet was low-carbohydrate (ie, no data provided on protocol diet carbohydrate content or reported dietary intake)

TABLE S7 (Continued)

Study #	ADA statement/recommendation	Citation: first author, year	Study type	Description	Participants	Duration	Findings for test diet	Comments
7		Davis NJ, 2009 [ref. 71, ADA 2014][73]	RCT	Compared the effects on weight loss and glycaemic control of a low-carbohydrate diet vs. low-fat diet in adults with T2D.	n = 105 overweight adults with T2D<br>91 (87%) completed study	1 yr	**For low-carbohydrate diet:**<br>◆ **HbA1C** at 3 mo. AR ↓0.64% (↓8.5%); at 1 y returned to baseline. BG nSS<br>◆ **HDL** AI ↑0.16 mmol/L (↑12.3%) BG SS<br>◆ **TRG, LDL** BG nSS<br>◆ **weight** both groups lost: AR ↓3.1 kg (↓3.4%) at 1 y<br>◆ **SBP, DBP, LDL, HDL, TRG:** No SS results<br>◆ **insulin use:** Reduced in low-carbohydrate arm and increased in control	**Should be included in a review of the evidence**<br>Does not support ADA statement 3. Does support statement 4
8		Daly ME, 2006 [ref. 112, ADA 2014][74]	RCT	Compared low-carbohydrate diet vs. reduced-portion, low-fat diet	**n =** 102 obese adults with poorly controlled T2D<br>79 (78%) completed study	3 mo	**For low-carbohydrate diet:**<br>◆ **HbA1c AR** ↓0.55% (↓6.1%) BG nSS<br>◆ **weight** AR ↓3.55 kg (↓3.5%) BG SS<br>◆ **Total cholesterol:HDL ratio** AR ↓0.48 (↓11.9%) BG SS<br>◆ **TRG** AR ↓0.67 mmol/L (↓27.0%) BG nSS<br>◆ **diabetes medication use:** Reduced more in low-carbohydrate arm vs control (insulin use ↓85% vs 22%)	**Should be included in a review of the evidence**<br>Partial support for ADA statements 2, 3 and 4<br>Note medication use
9		Dyson PA, 2007 [ref. 113, ADA 2014][75]	RCT	Assessed the impacts on body weight, HbA1c, ketone and lipid levels in diabetic and non-diabetic subjects, comparing low-carbohydrate diet vs. an E-restricted diet based on UK diabetes diet	n = 13 overweight or obese adults with T2D and 13 adults without T2D<br>12 (92%) with T2D completed study	3 mo	**For low-carbohydrate diet:**<br>◆ **HbA1c** AR ↓0.4%<br>↓**5.5%** BG nSS<br>◆ **weight** AR ↓8.0 kg BG SS<br>◆ **HDL, total cholesterol, LDL, TRG** BG nSS	**Should be included in a review of the evidence.**<br>Supports ADA statements 2, 4 and 5
10		Wolever TM 2008 [ref. 114, ADA 2014][76]	RCT	Aim was to compare the effects of altering the GI or the amount of carbohydrate on HbA1c,	162 adults with T2D<br>130 (80%) Completed	1 y	**HbA1c** at 1 year, was identical for the three diet groups	**Should be included in a review of the evidence**

TABLE S7 (Continued)

Study #	ADA statement/recommendation	Citation: first author, year	Study type	Description	Participants	Duration	Findings for test diet	Comments
				FBG, and other biomarkers.<br>Compared three diets: High-carbohydrate/ high-GI; high-carbohydrate/low-GI; lower-carbohydrate/ high-monounsaturated fat (39% carbohydrate)				Supports ADA statement 4
11		Kirk JK, 2008 [ref. 115, ADA 2014][77]	Systematic review/ meta-analysis	Reviewed 13 studies on carbohydrate-restricted diets for adults with T2D (RCT, crossover; RCT parallel; non-randomized two-arm;<br>Single-arm pre-post)	Adults with T2D. Study n range: 8--52.	1 wk to 26 wk	**HbA1c**<br>1 study ↑2.7%; 1 study 0% change; 9 studies ↓3.7%--22.4%<br>Of six studies with data for both low-carbohydrate and high-carbohydrate, low-carbohydrate performed better in four	**Should be included in a review of the evidence**<br>Some support for ADA statement 3
12		Iqbal N, 2010 [ref. 116, ADA 2014][78]	RCT	Compared effects of low-carbohydrate diet (30 g) vs. low-fat, calorie-restricted diet with<br>Low-intensity intervention	n = 144 OB adults with T2D<br>68 (47%) completed study	2 y	**HbA1c**<br>◆ at 6 mo AR ↓0.5% (↓6.3%) BG SS<br>At 2 y AR ↓0.1% (↓2.6%) BG nSS<br>◆ **FBG**<br>At 2 y AR ↓1.8 mg/dL ↓1.3%) BG nSS<br>◆ **weight**<br>At 2 y AR ↓1.5 kg ↓1.3% BG nSS<br>◆ **TRG** AR ↓26 mg/dL (16.8%) BG nSS<br>All other lipids nSS	**Should be included in a review of the evidence**<br>Supports ADA statement 5
13	"The role of low-carbohydrate diets in patients with diabetes remains unclear (72, ADA 2018)."	Wheeler ML, 2012 [ref. 72, ADA 2018][29]	Systematic review	2001–2010 review on diet and diabetes; included 11 studies on low-carbohydrate diets	n range = 10–55 per study group	2 wk to 1 y	◆ **HbA1c** decreased with a low-carbohydrate diet in 6 of 10 studies<br>◆ **FBG, 24-hour insulin, fasting insulin and insulin sensitivity** improved "significantly on the lower-carbohydrate diet."	**Should be included in a review**<br>Partial support for ADA statement note: Three of the reviewed studies reported carbohydrate consumption at

TABLE S7 (Continued)

Study #	ADA statement/recommendation	Citation: first author, year	Study type	Description	Participants	Duration	Findings for test diet	Comments
							◆ **need for diabetes medication** lower with lower-carbohydrate diets<br>◆ **lipids** some studies showed lower-carbohydrate diets improved lipid levels, mainly HDL and TRG<br>**Conclusion:** Evidence mixed and of not high quality due to study size, duration, dropout rates, or lack of randomization in some cases	<70 g/d; they attained an SS improvement in HbA1c of --6.8% to --17%
14	"While benefits to low-carbohydrate diets have been described, improvements tend to be in the short term and, over time, these effects are not maintained." (74–77, ADA 2018)	Snorgaard O, 2017 [ref.# 74, ADA 2018][79]	Systematic review and meta-analysis	Review/analysis of 10 RCTs to address the question: Is there an ideal amount of dietary carbohydrate for individuals with T2D? Analysed association of reported carbohydrate intake with reduction in HbA1c	Pooled n = 1376 adults with T2D	Varied	**Conclusion**:<br>◆ the ideal amount of carbohydrates in the diet in the management of T2D is unclear<br>◆ low-carbohydrate and moderate-carbohydrate diets have greater glucose-lowering effect compared with high-carbohydrate diets<br>◆ the greater the carbohydrate restriction, the greater glucose lowering<br>◆ apart from improvements in HbA1c over the short term, low-carbohydrate is not superior to high-carbohydrate for glycaemic control or weight	**Should be included in a review of the evidence**<br>Supports ADA statement
15		van Wyk HJ, 2016 [ref. 75, ADA 2018][80]	Review	Aimed to better understand efficacy of low-carbohydrate diets for glycaemic control as well as the reasons for different conclusions	Adults with T2D	≥ 4 wk	**Conclusions:** Variability in study design and subject characteristics, as well as reported carbohydrate intake, may account for differences in studies'	**Should be included in a review of the evidence, but with clarification**<br>Supports the ADA statement 9

TABLE S7 (Continued)

Study #	ADA statement/recommendation	Citation: first author, year	Study type	Description	Participants	Duration	Findings for test diet	Comments
				Among 9 meta-analyses on the subject.<br>Reviewed were 12 RCTs that were ≥ 4 weeks and which tested low-carbohydrate diets of ≤45 g/day,			findings; total E intake is the best predictor of body weight; low-carbohydrate diets perform no better than high-carbohydrate diets<br>To improve metabolic markers; very low-carbohydrate diets may not be sustainable, as carbohydrate intake often returns to a more moderate level over time	Note: It was likely adherence, not the low-carbohydrate diet *per se*, that determined outcomes.<br>Reported carbohydrate intake at end of study for low-carbohydrate arms, for the 12 studies, was 132--228 g/d
16		Meng Y, 2017 [ref. 76, ADA 2018][81]	Systematic review and meta-analysis	Reviewed 9 RCTs; aimed to assess the efficacy of low-carbohydrate diets compared to normal/higher-car bohydrate, low-fat diets in T2D	Pooled n = 734 adults with T2D	3- to 24 mo	◆ superior benefit for HbA1c, TRG, HDL, and short-term weight loss, but not LDL, total cholesterol, FBG or long-term weight loss, when compared to control diet	**Should be included in a review of the evidence**<br>Supports ADA statement<br>**Limitation:** Analysis did not consider reported carbohydrate intake of study diets but only target levels
17		Tay J, 2015 [ref. 77, ADA 2018][82]	RCT	Compared effects of a very low-carbohydrate, high-unsaturated fat/low-saturated fat diet vs. a high-carbohydrate, low-fat diet on glycaemic control and CVD risk factors in T2D<br>Both study arms E-restricted	n = 115 overweight or obese adults with T2D<br>78 (68%) completed study	1 y	◆ **HbA1c** AR ↓1.0% (↓13.6%) BG nSS<br>◆ **FBG** AR ↓0.7 mmol/L (↓9.0%) BG nSS<br>◆ **weight** loss AR ↓9.8 kg (↓9.6%) BG nSS<br>◆ **HDL** AI ↑0.1 mmol/L (↑8.3%) BG SS<br>◆ LDL AR ↓0.1 mmol/L BG nSS<br>◆ **TRG** AR ↓0.4 mmol/L (↓25%) BG SS<br>◆ **blood glucose stability**<br>SS improved with low-carbohydrate diet.<br>**Diabetes medication use:** Reduced more with low-carbohydrate diet. BG SS	**Should be included in a review of the evidence**

TABLE S7 (Continued)

Study #	ADA statement/recommendation	Citation: first author, year	Study type	Description	Participants	Duration	Findings for test diet	Comments
							Participants in the low-carbohydrate arm spend more time in euglycaemic range than hyperglycaemic range vs. those in high-carbohydrate arm	
18	"While some studies have shown modest benefits of very low-carbohydrate or ketogenic diets (less than 50-g carbohydrate per day (78,79), this approach may only be appropriate for Short-term implementation (up to 3–4 months) if desired by the patient, as there is little long-term research citing benefits or harm." (ADA 2018)	Goday A, 2016 [ref. 78, ADA 2018][83]	RCT	Evaluated the short-term safety and tolerability of a very low-carbohydrate, ketogenic diet (<50 g/d) in a weight loss/lifestyle modification programme for adults with T2D<br>VLCK group ate commercial weight-loss products and natural foods. Restricted-E control diet was based on ADA guidelines.	n = 89 obese adults with T2D<br>76 (85%) completed study	4 mo	**Safety**<br>No SS differences in safety parameters were found between the two study groups, including:<br>**Efficacy**<br>◆ **HbA1c** AR ↓0.9% (↓13.0%) BG SS<br>◆ **FBG** AR ↓28.0 mg/dL (↓20.5%) WG SS<br>BG nSS<br>◆ **HOMA-IR** AR ↓3.4 (↓49.3%) WG BG nSS<br>◆ **TRG** AR ↓35.9 mg/dL (↓23.9%) WG SS BG SS<br>◆ **HDL, LDL** no SS changes<br>◆ **weight** AR ↓14.7 kg (↓16.1%) BG SS<br>◆ **medications** significant decrease in low-carbohydrate arm only although no specific details on which medications were given	**Should be included in a review of the evidence**<br>Supports ADA statement in part; however, other studies (see table 8) provide safety and efficacy data from longer studies
19		Saslow L, 2017 [ref. 79, ADA 2018][84]	RCT	Compared effects on glycaemic control and other outcomes of two online interventions (ad libitum very-low-carbohydrate with behavioural support vs. ADA's "create your plate" diet	n = 25 overweight adults with T2D, 18 (72%) completed study	32 wk	◆ **HbA1c** AR ↓0.8% (↓11.3%) BG SS<br>◆ **weight** AR ↓12.7 kg (↓11.6%) BG SS<br>◆ **TRG** AR↓ 60.1 mg/dL (↓34.5%) BG SS<br>◆ **HDL, LDL** no SS changes	**Should be included in a** review of the evidence<br>Supports ADA statement in part; however, other studies (see table 8) provide safety and efficacy data from longer studies

Abbreviations: AHEI, alternate health eating index; AI, absolute increase; AR, absolute reduction; BG, between groups; ADA, American Diabetes Association; CV, cardiovascular; CVD, cardiovascular disease; DASH, Dietary Approaches to Stop Hypertension; DBP, diastolic blood pressure; DGA, Dietary Guidelines for Americans; E, energy; FBG, fasting blood glucose; HbA1c, glycated haemoglobin; HHS, Health and Human Services; HOMA-IR, homeostatic model assessment of insulin resistance; HR, hazard ratio; GI, glycaemic index; nSS, not statistically significant; OR, odds ratio; SS, statistically significant; RCT, randomized controlled trial; SBP, systolic blood pressure; TRG, triglyceride; T2D, type 2 diabetes; USDA United States Department of Agriculture; WG, within groups; VLCK, very low calorie ketogenic.
